# Multivariate Hawkes process models of the occurrence of regulatory elements

**DOI:** 10.1186/1471-2105-11-456

**Published:** 2010-09-09

**Authors:** Lisbeth Carstensen, Albin Sandelin, Ole Winther, Niels R Hansen

**Affiliations:** 1 The Bioinformatics Centre, Department of Biology & Biotech Research and Innovation Centre, University of Copenhagen, Ole Maaløes Vej 5, 2200 Copenhagen N, Denmark; 2 Department of Mathematical Sciences, University of Copenhagen, Universitetsparken 5, 2100 Copenhagen Ø, Denmark

## Abstract

**Background:**

A central question in molecular biology is how transcriptional regulatory elements (TREs) act in combination. Recent high-throughput data provide us with the location of multiple regulatory regions for multiple regulators, and thus with the possibility of analyzing the multivariate distribution of the occurrences of these TREs along the genome.

**Results:**

We present a model of TRE occurrences known as the Hawkes process. We illustrate the use of this model by analyzing two different publically available data sets. We are able to model, in detail, how the occurrence of one TRE is affected by the occurrences of others, and we can test a range of natural hypotheses about the dependencies among the TRE occurrences. In contrast to earlier efforts, pre-processing steps such as clustering or binning are not needed, and we thus retain information about the dependencies among the TREs that is otherwise lost. For each of the two data sets we provide two results: first, a qualitative description of the dependencies among the occurrences of the TREs, and second, quantitative results on the favored or avoided distances between the different TREs.

**Conclusions:**

The Hawkes process is a novel way of modeling the joint occurrences of multiple TREs along the genome that is capable of providing new insights into dependencies among elements involved in transcriptional regulation. The method is available as an R package from http://www.math.ku.dk/~richard/ppstat/.

## Background

Uncovering the details of the machinery involved in gene regulation remains an open problem in both experimental and computational biology. Part of this machinery is the collection of factors, along with the cognate transcription regulatory elements (TREs) that they bind to, that are responsible for the transcription of a given gene. This includes transcription factors and their sites, as well as histone modifications and other DNA-associated proteins. How these factors interact is to a large extent unknown. A fundamental problem in gene regulation bioinformatics is the limited information in the DNA binding typically displayed by transcription factors, which leads to many false positives when predicting binding sites in genomic sequences (reviewed in [1]). Since in vitro binding affinities can be accurately modeled using weight matrix models, the question is what additional information the cell uses to recruit the correct factor to its cognate sites. Combinations of sites will be more information-rich and indeed, there are combinations of sites (modules) that are responsible for tissue-specific gene expression, and which can also be used for prediction of regulatory regions [2,3].

Until recently, it was only possible to study the organization of binding sites for regulatory elements via computational methods, since experimental determination of single sites was time-consuming. Examples include [4], where cis-regulatory modules were detected by searching the promoters of co-expressed genes, and [5], where the authors constructed a genetic algorithm to learn the structure of the modules. These studies clearly showed that within modules, there are often preferred distances between binding sites [6]. However, while these methods have been successful, they cannot replace experimental methodology. Maturation of experimental techniques has made it possible to measure the binding of DNA-binding proteins over whole or partial genomes by e.g. Chromatin Immuno-Precipitation (ChIP) followed by sequencing, often called ChIP-seq [7], or ChIP followed by hybridization to DNA probes covering the genome, often called ChIP-chip [8]. These techniques open new avenues for analyzing gene regulation, and in particular interaction between regulators. Several of these kinds of data sets have been published. 

Despite the technical and experimental developments, we still lack a suitable multivariate model for the joint occurrences of multiple transcription factor binding sites and other TREs. Computational approaches generally only treat co-occurrence of sites in a pairwise manner. Pairwise analyses of the TREs, like the inter-motif distance analysis in [6], show that most TREs co-occur, but this tells us little about whether the co-occurrence is due to a direct relation between the two TREs or an indirect relation e.g. via other TREs. An observed pairwise co-occurrence might for instance be solely explained by the recruitment of the corresponding regulators by a third factor. For this reason, it is important to develop multivariate models to be able to detect whether observed relations are mediated through other TREs included in the model. 

To describe the phenomenon that TREs do not occur completely independently of each other, we will throughout this paper use the terms *interaction*, *relation *and *dependence*. Interaction is used to describe situations in which the combined occurrence of two TREs is important for a single regulatory purpose. Although the term has a physical connotation, we use it in a way that includes, but is not restricted to, physical interaction of the corresponding transcription regulators. Relation is a more vague term, which covers the general phenomenom of two TRE occurrences being somehow associated. Dependence is a statistical concept, and a relation between two TREs shows up as a dependence in their joint distribution of their occurrences. In the statistical models we will consider, we can make statements about dependence or independence - and in our case about a particular concept called local independence, [9]. The interpretation of the latter concept is that if two TREs are locally independent then there is no direct relation between the TREs. As the data considered in this paper are observational, we will not, however, be able to definitively deduce that any local *dependence *found in the data by our method represents a direct relation. To draw such conclusions, one would need stronger experimental evidence, for instance via an experiment activating and inactivating a particular TRE. For one of the data sets we analyze, there are experimental results supporting that some of our findings represent real interactions.

We analyze two data sets; for comparison, we review previous studies based on these data. The first data set is from Chen et al. [10] in which the locations of 13 sequence-specific TFs and 2 transcription regulators in mouse embryonic stem cells were mapped using the ChIP-seq method. The analyses for co-occurrences are based on what Chen et al. called multiple transcription factor-binding loci (MTL). These MTL were located by first finding peaks in the tags from the Chip-seq experiment and then iteratively clustering peaks that were close to one another. They found two groups of TFs that tended to co-occur. The first group consisted of Nanog, Sox2, Oct4, Smad1 and Stat3 and the second group consisted of n-myc, c-myc, E2f1 and Zfx. Some of the interactions between the TFs that were identified computationally were verified experimentally. Specifically, interactions between Oct4 and Smad1 and between Oct4 and Stat3 were verified. This was done by a depletion of Oct4, which led to a reduction in Smad1 and Stat3 binding at sites usually bound by Oct4 and Smad1 or Oct4 and Stat3, respectively. In addition, the association of p300 with Nanog-Oct4-Sox2 clusters was validated for 12 sites by using ChIP-qPCR. Depletion of Oct4, Sox2 or Nanog also reduced the binding of p300.

The second data set is from the ENCODE project [11]. This project aims to catalogue all functional regions of the human genome and is particularly important for the study of regulatory elements. In the pilot phase of the project, focusing on 1% of the genome, a large set of regulators were studied [12]. Several papers focusing on different types of analyses of data from this rich source have been published to date. The study by Zhang et al. [13] is worthy of mention, since it shares part of our scope: it is an exploratory study of the distribution of 29 different TREs assayed by different laboratories and under different experimental conditions, giving a total of 105 different ChIP-chip experiments. The main part of the analysis is based on 150 or 5 kb partitions, called 'genomic bins', of the regions analyzed. The distribution of ChIP-chip binding sites is quantified by counting the number of nucleotides, *M_ij_*, that each binding site, *i*, covers in each bin, *j*. The count matrix, *M*, for each ChIP-chip experiment is treated as the observation from the experiment and is then used in the correlation analyses. The purpose of this pre-processing step is to convert the positional data on TRE binding into matrix form, making the data amenable to standard multivariate analysis methods. Principal component analysis (PCA) and clustering analysis are then used to study the co-occurrence of TREs based on the genomic bins. The clustering provides a hierarchical grouping of the elements so that elements in the same group have a similar binding pattern as measured by the genomic bins. Likewise, PCA provides a picture, for instance via the bi-plot, of groupings in the data. The dominant correlation in the complete data set was ascribed to the effect of laboratory resulting in an Affymetrix and a non-Affymetrix subdivision of the data. For our analyses, we focus on the Affymetrix subset of the ChIP-chip data from the ENCODE pilot project. Interactions among the TREs in this set are to a large extent unknown.

While correlation analyses based on MTLs or genomic bins might provide insights into occurrences of sites, we have some concerns with these approaches. Most importantly, the choice of the clustering distance in the case of MTLs and the genomic bin size limit the analyses to dependencies that are compatible with the chosen scale. In particular, all specific details of dependencies within the locus or genomic bin are lost. In addition, the binning is initiated at an arbitrary starting point and the choice of starting point could affect the results obtained; in other words, the placement of the bins might affect the final result. Consequently, the correlation analyses may not provide a complete picture of how correlated TREs affect one another's occurrences. Moreover, the analyses in [13] focused on correlation, which in reality is a matter of analyzing pairwise co-occurrences. Steps were not taken to unravel the intricate details of the multivariate distribution of TRE occurrences. Intuitively, statistical models chosen to understand biological processes should be fitted to the biological data at hand, rather than the reverse. We suggest that the multivariate Hawkes point process model is a more suitable framework for analyzing TRE data, since no clustering of sites or binning is necessary.

Our main result is to show that multivariate point process models - and in particular the Hawkes process - are suitable for analyzing TRE occurrences. Moreover, we provide detailed results of separate analyses of the distribution of eleven TREs from Chen et al [10] and eight TREs from the Affymetrix ChIP-chip experiment on the pilot ENCODE regions. We identify the TREs that do not show direct relations with other TREs, and present quantitative results as to how much the occurrence of one TRE affects the probability of the occurrence of other TREs, as a function of the distance between them. Notably, in our analysis of the data from mouse embryonic stem cells, our method yields quantitative conclusions similar to those in [10]; we find the experimentally verified interactions and detect the same grouping of the TFs as the original study. Additionally, our model provides more detailed quantitative information and we detect an interaction between two TFs that was missed by the analysis in [10] but was experimentally verified in other studies [14].

## Results

The objective is to investigate whether the occurrence of one TRE directly affects the occurrences of other TREs. The correct scale for studying the organization of TREs on the genome seems to be a scale where most regulators show point-like interactions with the genome at binding sites that each cover only a few nucleotides, since this corresponds to actual binding site sizes. At this stage, it is helpful to review the ChIP-seq and ChIP-chip techniques. ChIP-seq/chip are based on a protocol that first fixes DNA-bound proteins to DNA by cross-linking, followed by shearing of the DNA. Antibodies are then added to isolate the DNA bound by a protein of interest (see [15] and references therein for an introduction). The sheared DNA sequences are approximately 400-1000 bp long, depending on the protocol used. For ChIP-seq, the 5' or 3' edges of the fragments are sequenced. These sequence reads can then be mapped back to the genome, and the site of the cognate DNA-binding protein can be determined using specific algorithms, see e.g. [16]. Adjacent regions on the DNA with a high frequency of mapped sheared sequences from the ChIP step are merged into a larger block. This implies that it is possible to merge two or more binding sites in a single block, since individual signals end up being merged if they are physically close together. For ChIP-chip, the sheared DNA sequences are hybridized to an array of DNA probes that are designed to cover the genome with a certain spacing between the probes. As for ChIP-seq data, sets of probes that are consecutive on the genome and have high hybridization signals will be merged into a larger block corresponding to the DNA region spanning the probes. Since probes cannot be placed on repetitive genomic sequences and repetitive sequences may map to more than one location on the genome, a larger block can be broken up by occurrences of repeat elements in both ChIP-seq and ChIP-chip data. Finally, since both methods are based on a ChIP step, the regions detected are much larger than the actual underlying TREs, and hence we need to obtain proxies for the actual locations of the TREs from the wider ChIP data.

**Figure 1:**
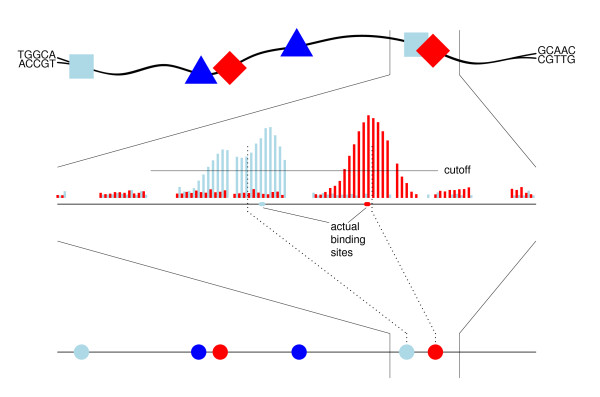
**ChIP-chip to point process**. Illustration of the way in which data from the ChIP-chip experiment can be viewed as a point process. In each cell, the different TREs are positioned along the double stranded DNA-sequence (top). The abundance of binding sites across cells at a particular position of the sequence results in a signal generated from the ChIP-chip experiment (middle). The midpoint of the interval where the signal is above a specified cut off is used as a proxy for the actual binding site. The midpoints for each of the TREs considered are viewed as points from a multivariate point process along the line (bottom).

Figure 1 illustrates how we obtain proxies for binding sites for at given TRE from a ChIP-chip signal. For ChIP-seq data the approach is essentially identical, although the raw signal is slightly different. When considering several TREs, we ultimately obtain a sequence of points along the genome with different labels according to the different TREs, i.e., a multivariate point process along the genome. The intensity of occurrence of one TRE along the genome is then affected by the occurrences of the other TREs, and so we need to model how this intensity changes along the genome and how the occurrences of other TREs affect the intensity.

### Multivariate analysis of TREs for mouse embryonic stem cell data

For the application of our model to the ChIP-seq data from Chen et al [10], we focus on 11 TREs: 9 TFs and 2 transcription regulators. We let

I={Nanog, Oct4, Sox2, Stat3, Smad1, Zfx, c-myc, n-myc, E2f1, p300, Suz12}

denote the set of these TREs. Inspired by [17], we investigate the use of the Hawkes process, where the main specification consists of a collection of functions *g_m, k _*for each combination of *m*, *k *∈ *I *of TREs considered (see Methods). Given that we observe TRE *m*, the intensity for observing TRE *k *downstream at a distance s is then given as a baseline intensity multiplied by *g_m, k_*(s). The most important hypotheses to investigate are whether *g_m, k _*= 1, which means that the occurrence of TRE *k *is not affected by upstream occurrences of TRE *m*. We can use standard methods for testing these hypotheses and as described in the Methods section, we can use the results to determine local independencies in the data. 

In the multivariate analysis of the 11 TREs we initially allow for all 121 potential interactions among the TREs. As described in the Methods section, each of the *g*-functions are modeled using a spline basis expansion of log *g_m, k _*for *m*, *k *∈ *I *with 8 equidistant, fixed knots. We choose to place the knots so that we limit the range of dependence to a maximum of 1000 base pairs downstream.

**Figure 2:**
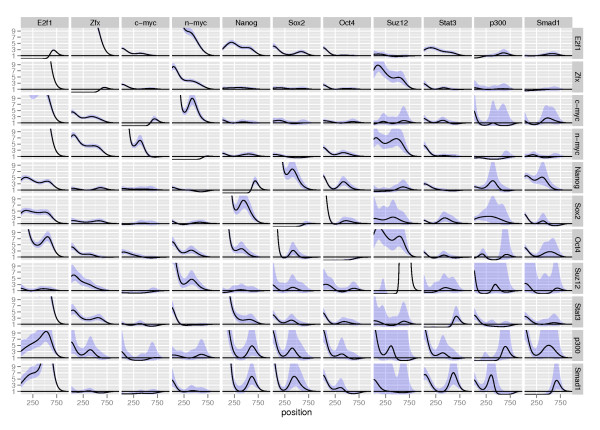
**Estimated ***g***-functions, forward direction, mouse embryonic stem cell data**. Plots of the *g*-functions modeling the effect of the occurrence of one TRE (column) on the occurrence of another TRE (row). The effects are estimated in the multivariate model. A value less than one indicates that this inter-TRE distance tends not to occur while a value greater than one indicates an inter-TRE distance that is likely. Point-wise 95% confidence intervals for the functions are also shown. To ease comparisons between effects, all the y-axes have the same scale with a maximum value of 10.

Estimates of the 121 *g*-functions are shown in Figure 2. For each TRE(column), these functions show the factor by which that TRE affects the downstream baseline intensity of another TRE (row), as a function of the distance between the TREs. A point-wise 95% confidence interval is also shown for each of the estimated functions. Adopting the terminology of [17] we say that a value less than one means that a given inter-TRE distance tends to be avoided, while a value greater than one means that a given inter-TRE distance tends to be favored.

It is important to point out that the implementation of the Hawkes process treats the genome simply as a line along which events (the occurrences of TREs) happen. This means that the descriptors "downstream" and "upstream" are dependent only on the direction we assign to the genome and not on the actual direction of genes. The estimated *g_m, k_*-functions in Figure 2 were estimated in the forward direction of the genome; i.e., the lowest numbered nucleotide in each chromosome based on the assembly coordinates was used as the starting point. We will discuss this point in more depth below.

The overall impression from Figure 2 is that generally, the occurrence of one TRE affects the occurrence of another TRE by increasing its intensity immediately downstream, with the effect then leveling off. For instance, there is a more than 10-fold increase in the intensity for occurrences of Zfx, c-myc and n-myc immedialy downstream of E2f1. However, several of the estimated effects do not seem to be significantly different from one.

The largest positive effects are found among the four TREs in the upper left corner, E2f1, Zfx, c-myc and n-myc and among the three TREs Nanog, Sox2 and Oct4. This indicates that the factors in the two groups often bind in proximity to each other. In addition, the three TREs Stat3, p300 and Smad1 seem to be more related to the group consisting of Nanog, Sox2 and Oct4 than to the other group. This is consistent with the analyses by Chen et al [10]. We also observe possible relations between Suz12 and Oct4 and Zfx and n-myc, respectively. In the original study, no relation with Suz12 was reported, but another study reported an interaction between Suz12 and Oct4 in mouse embryonic stem cells [14]. The experimental validation was based on both PCR analysis of the promoters and a knockdown study where reduced levels of Oct4 led to a loss of Suz12 at certain target promoters.

**Figure 3:**
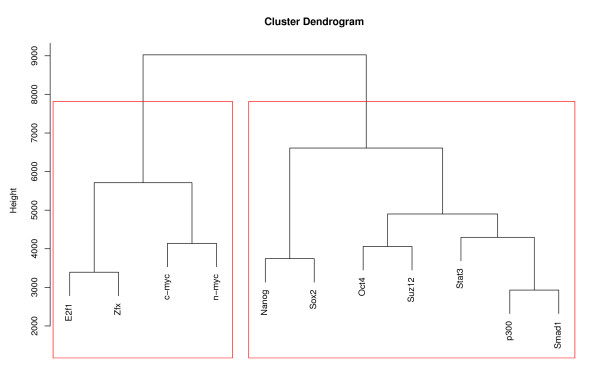
**Clustering of TREs based on interaction graphs, mouse embryonic stem cell data**. Result of a hierarchical clustering procedure based on the Ward method of the graphs for each TRE given in Figure 2. The clustering is based on the integral of the absolute value of the logarithm of the functions in Figure 2.

Based on the log *g_m, k_*-functions, we clustered the 11 TREs (see Methods) in order to link regulators that co-occur. The result of this clustering is presented in Figure 3 and shows two clusters of TREs. The first cluster consists of E2f1, Zfx, c-myc and n-myc, while the second includes Nanog, Sox2, Oct4, Suz12, Stat3, p300 and Smad1. Again, this is consistent with the results presented in [10], apart from the Suz12 findings. 

The functions on the diagonal (from upper left corner to bottom right corner) in Figure 2 represent the self-dependencies of the TREs, i.e., the effect of the occurrence of a TRE on the downstream occurrence of that same TRE. All 11 effects seem significantly different from one and all have a characteristic shape, with a clear depletion of the intensity for the first 500 nucleotides. For some of the TREs, the effects approach 1 thereafter and for others, there is a small positive effect after 500 nucleotides. The self-dependence for the transcription regulator Suz12 stands out, with a large (more than ten-fold) effect after 500 nucleotides. While there are many reports that show homotypic clusters of transcription factor binding sites [18,19], the depletion and peaks might be technical artifacts. Depletions can be attributed to the case where multiple binding sites are located very close to each other; in this case, the block will be interpreted as a single binding event, with the implication that sites located close together will never be detected. Peaks might occur due to the presence of repeat elements that break up larger regions. In the Suz12 case, the effect after 500 bp does not seem to be a technical artifact, since the effect is large.

In most cases, transcription factors have no strand preference relative to their regulated gene when it comes to binding, and regardless, strand information is lost in the ChIP experiment and the experiment will not explicitly tell us which gene is the regulatory target of a given site. Consequently, we fit the model to a mixed signal. If two TREs typically occur in a specific order when involved in the regulation of a gene, then the order is reversed from the forward direction point-of-view if the TREs are involved in the regulation of a gene in the reverse direction. We argue that if there is an equal distribution of TREs involved in regulation in the forward and reverse directions, the mixed signal should be approximately symmetric, which would then imply that the shapes of *g_m, k _*and *g_k, m _*do not differ that much up to a multiplicity factor. From Figure 2, we see that this is true in most cases (e.g. *g*_E2f1, Zfx _and *g*_Zfx, E2f1_) but we also see some deviations from this (e.g. *g*_Nanog, Smad1 _and *g*_Smad1, Nanog_).

To further investigate the estimated effects for each combination of *m*, *k *∈ *I*, we test the hypothesis

$$H_{0}\left(m,k\right):g_{m,k}=1.$$

**Figure 4:**
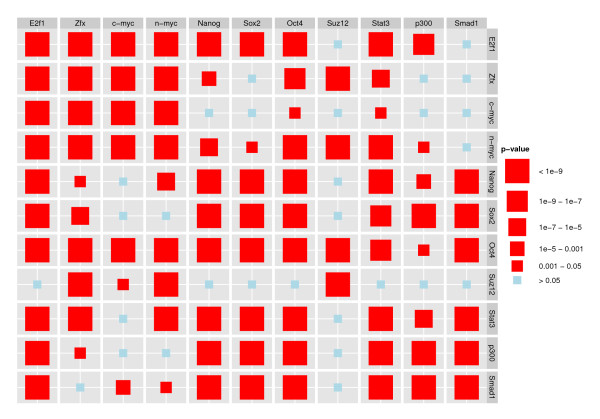
**Tests for local independence, mouse embryonic stem cell data**. This figure shows results for the 121 parallel likelihood ratio tests for local independence between all pairs of the 11 TREs in the multivariate model. We show the results for the model estimated in the forward direction (squares, effect of TRE (column) on TRE (row)). The size of the symbol for each test corresponds to the magnitude of the test statistic. Correcting for multiple testing using Holm's procedure the hypotheses of local independence that are rejected are shown in red while the hypotheses that are not rejected are shown in blue.

This is the hypothesis of local independence of the *m*'th TRE on the *k*'th TRE, conditional on the upstream occurrences of the *k*th TRE and the remaining 9 TREs, as described in the Methods section. If we reject *H*_0_(*m*, *k*), the interpretation is that there is a direct relation between the occurrence of TRE *m *and the downstream occurrence of TRE *k*. Of course, we can not rule out that such a relation can be explained by a factor not included in our analysis, but we can say that the other TREs included can not collectively explain the relation. On the other hand, if we do not reject *H*_0_(*m*, *k*), there is no evidence in the data for a direct relation between the occurrence of TRE *m *and the downstream occurrence of TRE *k*. The *p*-values for the 121 tests are shown in Figure 4, with tests for which *H*_0 _is rejected shown as red squares. As in Figure 2, we use the forward direction of the genome. We find that all TREs show significant self-dependence, as discussed above. In addition, we observe that many of the interactions are significant, with Oct4 having interactions with all the other TFs, and Suz12 having the fewest interactions with other TFs. All interactions within the group consisting of E2f1, Zfx, c-myc and n-myc are significant, as is those within the group consisting of Nanog, Sox2, Oct4, Stat3, p300 and Smad1, which again is in accordance with the analyses in [10].

We found a significant effect of the occurrence of Suz12 on downstream occurrences of Oct4 but not the reverse. We argue that this asymmetry is a consequence of the inclusion of self-dependence terms in the model, combined with Suz12's strong self-dependence, as can be seen in Figure 2. When we fit a model without the self-dependence term for Suz12 (not shown), the asymmetry disappears. We believe that most of the observed self-dependencies, including the large self-dependence for Suz12, represent true self-dependencies; therefore we prefer to use the multivariate model including all self-dependence terms when analyzing the data.

### Multivariate analysis of TREs for the pilot ENCODE regions

To further investigate the applicability of our model we analyze a subset of the ENCODE pilot data produced by Affymetrix: the "Affymetrix Sites" track from the UCSC ENCODE browser database resulting from a study of retinoic acid-treated HL-60 cells 0, 2, 8 and 32 hours after treatment. Initially, we focus on the 8-hour post-treatment results from the data and investigate the effects of the oriented specification of the model and the inclusion of histone modifications in the model. Subsequently, we compare the results obtained at the four different time points. We focused on 10 TREs, selecting classical transcription factors, the transcription machinery and chromatin boundary elements. Because some regulatory elements, such as histone modifications, can not always be regarded as point-like, we include the two histone modifications, H3K27me3 and H4Kac4, as covariates in the modeling of the remaining eight TREs. We let

I={BRG1, CEBPE, CTCF, P300, POL2, PU1, RARA SIRT1}

denote the set of these TREs. Aside from the inclusion of the histone modifications, an important feature, the model is the same as in the previous section. The intensity of the occurrence of a TRE at a given location depends on upstream occurrences of other TREs and on whether the histone modifications are present at the same location.

The set of TREs available from the ENCODE data is quite diverse and potential interactions among them are to a large extent previously undescribed. In the multivariate analysis of the 8 TREs, we initially allow for all 64 potential dependencies among the TREs. Again, as described in the Methods section, each of the *g*-functions are modeled using a spline basis expansion with 8 equidistant, fixed knots and a maximum range of dependence of 1000 base pairs downstream. (We did not seem to be able to capture dependencies over a longer range).

**Figure 5:**
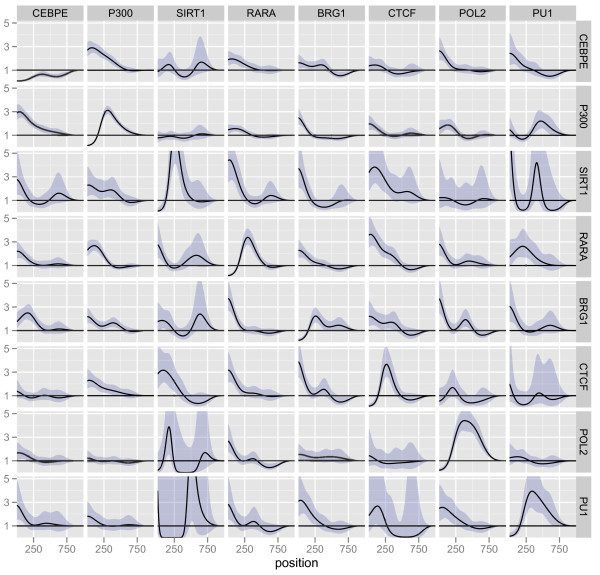
**Estimated ***g***-functions, forward direction, ENCODE data**. Plots of the *g*-functions modeling the effect of the occurrence of one TRE (column) on the occurrence of another TRE (row). The effects are estimated in the multivariate model adjusting for the histone modifications and allowing different baseline intensities for the ENCODE regions. A value less than one indicates that this inter-TRE distance tends not to occur while a value greater than one indicates an inter-TRE distance that is likely. Point-wise 95% confidence intervals for the functions are also shown.

Estimates of the 64 *g*-functions are shown in Figure 5, with the effect of each TRE (column) on the downstream baseline intensity of another TRE (row) as a function of the distance between the TREs. As with the previous data set, we observe that several TREs show a clear impact on the downstream occurrence of other TREs and, as in the previous analysis, most estimated effects seem greater than one, suggesting that in general, the occurrence of a given TRE affects the occurrence of another TRE by increasing its intensity. All 8 self-dependencies are significantly different from one, and for all except CEBPE, the *g*-function have a shape with a depletion of the intensity for the first 100 nucleotides, followed by a peak, with, for instance, a four-fold increase of the intensity for POL2 at approximately 300 nucleotides downstream. CEBPE shows a different behavior with only the depletion of the intensity downstream of the occurrence of CEBPE. As noted previously, both depletions and peaks might be technical artifacts. For ChIP-chip data, the peaks can be caused by genomic repeats, which can break up a longer signal block, causing the block to be interpreted as two binding events. On the other hand, the fact that CEBPE does not show the same behavior indicates that the effect may actually not be a technical artifact. Depletions might be attributed to multiple binding events occurring very close to one another, such that the block is interpreted as a single binding event, and since we use the center of the block as our proxy for the binding site, a depletion will occur if the block is large.

### Investigation of the oriented specification of the model

**Figure 6:**
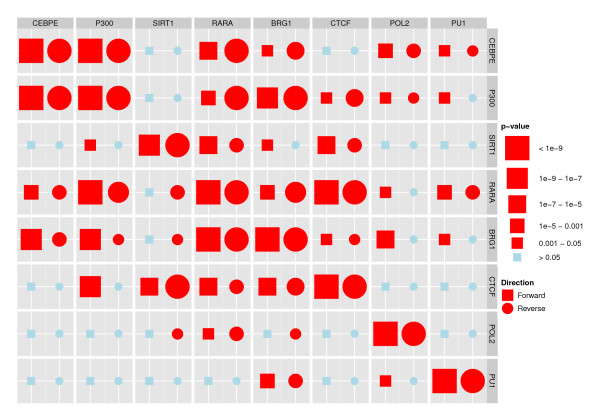
**Tests for local independence, ENCODE data**. This figure shows results for the 64 parallel likelihood ratio tests for local independence between all pairs of the 8 TREs in the multivariate model adjusting for histone modifications and different baseline intensities. We show the results for the model estimated in the forward direction (squares, effect of TRE (column) on TRE (row)) as well as in the reverse direction (circles, effect of TRE (row) on TRE (column)). The size of the symbol for each test corresponds to the magnitude of the test statistic. Correcting for multiple testing using Holm's procedure the hypotheses of local independence that are rejected are shown in red while the hypotheses that are not rejected are shown in blue.

To investigate whether the estimated effects are statistically significant, for each combination of *m*, *k *∈ *I *we test the hypothesis *H*_0_(*m*, *k*): *g_m, k _*= 1. The *p*-values are shown in Figure 6, with tests for which the hypothesis is rejected shown as red squares. As in Figure 5, we use the forward direction of the genome. We find that all TREs show significant self-dependence, as discussed above. We observe that RARA appear to be directly associated with most of the other TREs, both up- and downstream, whereas PU1 and POL2 show fewer significant direct relations with other TREs.

Keeping in mind that we fit the model to a mixed signal, we would expect to reject the hypothesis *g_m, k _*= 1 if, and only if, we reject the hypothesis *g_k, m _*= 1. Deviances from this, as seen in Figure 6, could be explained by an unequal distribution of these TREs involved in regulations in the forward and reverse directions, respectively, in this data set.

**Figure 7:**
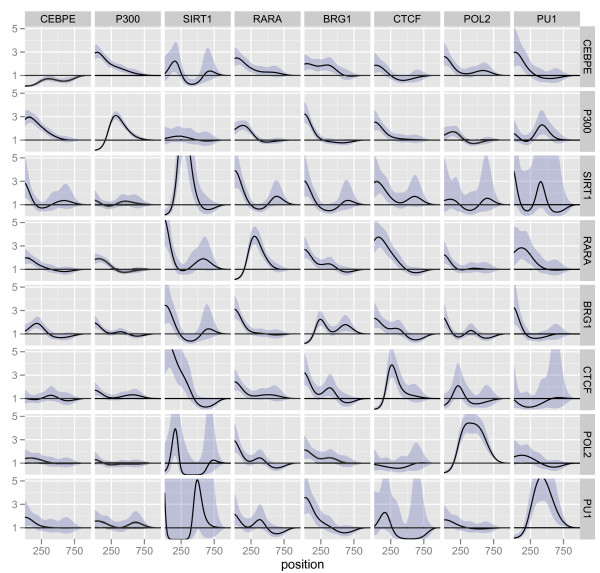
**Estimated ***g***-functions, reverse direction, ENCODE data**. Plots of the-functions modeling the effect of the occurrence of one TRE (row) on the occurrence of another TRE (column), estimated in the reverse direction. Note that the figure is transposed compared to Figure 5. The effects are estimated in the multivariate model adjusting for the histone modifications and allowing for different baseline intensities for the ENCODE regions. A value less than one indicates that this inter-TRE distance tends not to occur while a value greater than one indicates an inter-TRE distance that is likely. Point-wise 95% confidence intervals for the functions are also shown.

Regardless of a mixed signal, if we fit the model using the reverse direction of the genome, we would expect the estimate of *g_k, m _*to be similar to the estimate of *g_m, k _*in the forward direction. The *g*-functions estimated in the reverse direction are shown in Figure 7. To make the comparison with Figure 5 easier, for each TRE (row), the figure shows the factor by which that TRE affects the downstream baseline intensity of another TRE (column); with this figure orientation the estimate of *g_k, m _*in the reverse direction is in the same place in Figure 7 as the estimate of *g_m, k _*in the forward direction in Figure 5. We see that the estimated functions are indeed very similar for almost all of the TREs, with most differences being differences in function amplitude only and not in shape. The different amplitudes can be due to TREs occurring at different rates, such that at least one of the TREs will occur in situations without the other TRE.

We would also hope that the conclusions reached would be qualitatively symmetric - that is to say, that we reject *H*_0_(*m, k*) in the forward direction if, and only if, we reject *H*_0_(*k*, *m*) in the reverse direction. When we repeat the tests fitting the model in the reverse direction, we find that this is generally, but not entirely, the case. The *p*-values are shown in Figure 6 with tests for which *H*_0 _is rejected shown as red circles. As in Figure 7, we have transposed the results relative to the forward direction, that is, the figure shows the effect of the TRE (row) on the TRE (column), to make the comparison simple. Qualitatively, most of the conclusions are preserved, but 12 of the 64 tests had a different outcome when we estimate the model in the reverse direction. Most notably, BRG1, POL2 and SIRT1 are each involved in 5 of these tests. However, the conclusions for CEBPE are identical and for CTCF, there is only one difference.

An explanation for seeing a direct relation of TRE *m *on downstream occurrences of TRE *k *in the forward direction, but not a direct relation of TRE *k *on downstream occurrences of TRE *m *in the reverse direction, could be that TRE *m *occurs almost exclusively in relation to TRE *k*, but TRE *k *also occurs in many other situations in the absence of TRE *m*. This could explain the results for the relations between BRG1 and POL2 and between SIRT1 and BRG1, since in these cases we see a direct relation of TRE *m *(POL2 and BRG1, respectively) on the downstream occurrences of TRE *k *(BRG1 and SIRT1, respectively) in the forward as well as in the reverse direction.

In conclusion, certain findings are consistent when estimating in either direction. The self-dependencies are all significant, and RARA seems to have direct relations to all or most of the other TREs both up- and downstream. RARA is known to function as an active repressor by recruiting corepressors and/or deacetylases when its ligand is not present, and an activator when the ligand is present [20]. Our observation that RARA is directly related to most of the other TREs is then an indication of the importance of the factor in this system, since the cells were treated with its ligand (retinoic acid-treated HL60 cells).

### Effect of histone modifications on occurrences of TREs in the pilot ENCODE regions

As mentioned above, the two histone modifications are included as covariates in our model. The effects of the histone modifications on the intensity for the occurrence of TRE *k *in a modified region is captured by the fold change parameters $\gamma_{\text{H}3\text{K}27\text{me}3}^{k}$ and $\gamma_{\text{H}4\text{Kac}4}^{k}$, respectively (see Methods).

**Figure 8:**
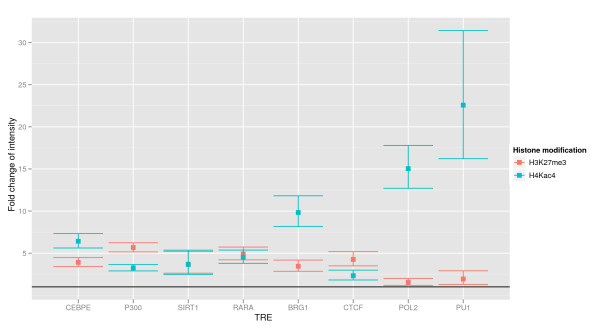
**Effect of histone modifications, ENCODE data**. Estimates and 95% confidence intervals for the parameters $\gamma_{\text{H}4\text{Kac}4}^{k}$ and $\gamma_{\text{H}3\text{K}27\text{me}3}^{k}$ for *k *one of the eight different TREs considered. These factors give the fold-changes of the baseline intensity in the presence of one of the histone modifications for each of the eight TREs.

Figure 8 shows the point estimates and 95% confidence intervals for these parameters. We find that all parameters are significantly greater than 1, showing that the intensity of the occurrence of any of the 8 TREs is increased in the presence of either of the histone modifications. Most notable is the effect of H4Kac4 on PU1 and POL2; the presence of H4Kac4 increases the intensity of the occurrence of these two TREs by a factor of 22.2 and 16.6, respectively. It is generally believed that acetylation is coupled to chromatin opening and increased transcription; indeed, an independent report shows a characteristic pattern of acetylation around transcription start sites [21]. However, it is not clear why the effect on PU1 and POL2 is so strong. One possible explanation is that the PU1 binding is not particulary stringent - it is a HMG box which essentially binds GGAA-rich sequences.

The effect of H3K27me3 on the occurrence of PU1 and POL2 is, on the other hand, negligible, which might have to do with its effect as a repressor [22]. We observe that the presence of H4Kac4 also show a pronounced effect on the occurrence of BRG1 and CEBPE, increasing the intensity by a factor of 9.9 and 6.2, respectively. The most pronounced effects of the presence of H3K27me3 are found for P300, RARA and CTCF where the intensity is increased by a factor of 5.8, 4.7 and 4.5, respectively.

### Results for all four time points for the pilot ENCODE regions

**Figure 9:**
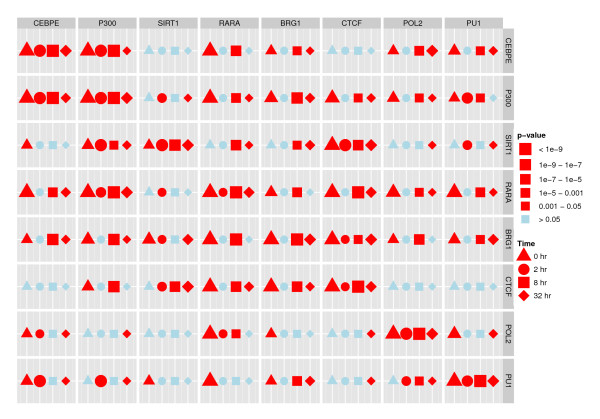
**Tests for local independence, all four time points, ENCODE data**. This figure shows the results for the 64 parallel tests for local independence between all pairs of the 8 TREs in the multivariate model, adjusting for all covariates, at the four time points (0, 2, 8, 32 hours post-treatment). The models are estimated in the forward direction with the effect of TRE (column) on TRE (row). The size of the symbol for each test corresponds to the magnitude of the test statistic. Correcting for multiple testing using Holm's procedure the hypotheses of local independence that are rejected are shown in red while the hypotheses that are not rejected are shown in blue.

Since data are available for 0, 2 and 32 hours post-treatment, in addition to the 8 hours analyzed initially, we can investigate whether our findings are consistent over time. Hence we fit our multivariate model to the data at these time points in the forward direction and test the hypotheses *g_m, k _*= 1 for all *m*, *k *∈ *I*. The *p*-values are shown in Figure 9. We see that the results for 2 hours after treatment stand out compared to the other three time points, with many fewer significant relations. BRG1 especially shows almost no relations with the other TREs, with the effect of CEBPE on downstream occurrences of BRG1 the only exception. In particular, we note that the self-dependence has disappeared. In contrast, BRG1 shows many relations with other TREs at the other three time points. The fact that BRG1 is observed less frequently 2 hours post-treatment compared with the other time points, combined with inspection of the estimated *g*-functions and their 95% confidence intervals (not shown), suggest that the much larger variance in the estimated signal for BRG1 relations 2 hours post-treatment are at least part of the explanation for the observed differences among the time points. Apart from the observations 2 hours post-treatment, many of the relations are consistent over time. We observe, for instance, that the local independence of CEBPE and CTCF is present at all four time points, as is the relation between CEBPE and P300. We should note, however, that although many relations are preserved over time, this does not necessarily mean that the binding sites for the TFs are the same over time, only that there is a relation between the binding sites for the TFs.

## Discussion

The analyses presented here for the Pilot ENCODE data are based on a single set of ChIP-chip data from the Pilot ENCODE project, which only covers 1% of the genome. The interactions between the factors in this set have not been verified experimentally to date. This means that the findings from our analysis should be interpreted with some caution, in particular when extrapolating them to the whole genome. As with all computational methods, experiments are needed to verify that specific interactions are significant; the role of computational analyses is to give good starting points for experimental studies. However, our analysis of the genome-wide ChIP-seq data from mouse embryonic stem cells shows that our method is able to identify interactions that can be verified experimentally. Moreover, the estimated *g*-functions provide interpretable, quantitative information on how the different TREs interact. Furthermore, this quantification can be used to cluster the TREs; however, we do not wish to overemphasize the applicability of this procedure in its current form, as we found the results of the clustering to be sensitive to the input data and choice of method.

Ultimately, the goal is to understand the causal relations among the many components involved in the regulation of gene expression. Our analysis provide a step in that direction but, as always with statistical analyses of observational data, we can not prove that an observed direct relation is causal - and even if it is, the analysis can not show the direction of the causality. To draw such conclusions, we need either experimental data or stronger causal assumptions [23].

A major contribution of our multivariate analysis consists of the collection of local independencies among the TREs that we identify and which would not have been revealed using pairwise methods. Our analysis enables us to say which TREs that do not seem to interact in the regulation of genes, allowing subsequent experimental studies to be focused on other combinations of TREs. To illustrate this point, we observe in Figure 4 that Oct4 has a significant relation to upstream occurrences of Suz12, but that Suz12 and Sox2 show no significant relations. Oct4 and Sox2 do, however, show significant relations. A pairwise analysis including only Suz12 and Sox2 results in a significant relation between the two (not shown), but in light of the results of our multivariate analysis, we interpret this as an indirect effect mediated through Oct4. 

The Hawkes process is not the only suitable model for these data. The main reason for focusing on the Hawkes process is that it is a flexible class of models, and the specification of the model as given in the Methods section allows us to compute the likelihood function directly, such that we can easily apply standard methods (maximum-likelihood estimation and likelihood-ratio tests). It might be argued that a drawback of the model is that we can only include information about upstream events in the conditional specification of the Hawkes process. However, the specification is a purely technical matter that does not by any means rule out the possibility of the Hawkes process capturing relations in both the up- and downstream directions, as seen from the comparison of results from the analysis in the forward direction with those from the analysis in the reverse direction. If we were to use spatial point process models, it would be possible to specify models that have no directionality in their specification. However, we have showed that most of the conclusions we obtain from the analysis using the Hawkes process are robust to the direction we use for estimation. A range of point process models, including the Hawkes process are used to model financial trading data [24]. The literature on spatial point processes is also rich, see e.g. [25], and although most of these models were developed for two or three dimensions, they are also perfectly applicable in a one-dimensional setup. Even so, for the typical spatial point process models the statistical inference becomes more subtle than for the Hawkes process.

It can be argued that the choice of knots, and hence the number and range of the spline basis functions used in the specification of the Hawkes process, could affect the results obtained. We used a relatively small number of knots and a relatively narrow range, but although details might change had we used more sophisticated knot positioning strategies, we found that our results, the qualitative conclusions in particular, were robust to the actual choice of knots.

We illustrated the use of the Hawkes process with analyses of ChIP-chip/seq data for TREs but the model can be equally useful for other types of multivariate, positional genome data, whether these data are experimental or computational. Examples of such data are transcription factor binding sites, small RNAs, or even genetic polymorphisms in different individuals. Compared with the use of alternative methods such as in [10] and [13], where clustering of sites or genomic binning are needed, no such pre-processing steps are necessary for the application of the Hawkes process. The model can capture both short- and long-range dependencies. With genomic binning, potential dependencies over longer distances are ignored in [13], and the fine details of short-range dependencies are lost by the methods used in both [10] and [13]. Finally, we note that our method is based on a generative model on the scale of binding sites, in contrast to the models considered in [10] and [13].

## Conclusions

We have presented a statistical method to analyze the multivariate distribution of TREs along the DNA sequence. We have shown that by using the point process approach, we can perform a detailed analysis of the multivariate distribution of TREs, providing both insightful qualitative information about local independence among the TREs and quantitative information on how the TREs affect the occurrence of one another. Furthermore, we have shown that our method is able to detect experimentally verified interactions, as well as interactions missed by other computational methods. We find that to understand the interactions among many TREs, it is crucial to carry out the analyses in a multivariate framework that includes all available information and relevant covariates; such an analysis emphasizes direct relations rather than indirect relations among the TREs investigated.

## Methods

### Mouse embryonic stem cell data

The analysis of the core transcriptional network in mice embryonic stem cells presented in [10] is mainly based on ChIP-seq data for 13 sequence-specific TFs and 2 transcription regulators. We analyze the ChIP-seq data with a focus on 9 of the TFs as well as the 2 transcription regulators. For our analysis, we use the data from [10] given in the supplementary material, Additional file 1 and 2, taking the midpoints of the enriched regions as the binding sites. In cases where the midpoint is between two base pairs, we take the lesser of these as the midpoint. We choose to restrict our analysis to the 19 autosomes, since the distribution of TREs on the X sex-chromosome appears different from the distribution of TREs on the other 19 chromosomes.

### ENCODE data set

In this analysis, we consider ChIP-chip data produced by Affymetrix for the ENCODE pilot project as given the supplementary material, Additional file 3, 4, 5, 6, and 7. In Additional file 8 the data is illustrated. The data contains regions with locations of 10 different TREs in retinoic acid-stimulated (human) HL-60 cells harvested 0, 2, 8, and 32 hours after treatment. This provides us with data from cells in the same stage of the cell cycle and hence the ChIP-chip data yields information about regulatory elements bound to the DNA sequences at the same time.

The ChIP-chip regions, which in this study have a mean length of approximately 400 base pairs, are regions of DNA enriched with a regulatory element. To model the binding site locations from the ChIP-chip experiments as a point process, we choose the midpoints of the ChIP-chip signals as the binding sites (see Figure 1). As for the mouse embryonic stem cell data, in cases where the midpoint is between two base pairs, we take the lesser of these as the midpoint.

The two histone modifications enter as covariates in the model; in this case, we choose to use the whole enriched sequence by including them as indicator functions.

### Point processes

A point process is a model for points or events that occur randomly in time and/or space. Here, we consider point processes for points occurring along the DNA sequence, i.e., points that can be represented on a one-dimensional line. We assume that no more than one point occurs at the same location, yielding a simple point process. Points of interest along the DNA sequence will typically be the locations of TREs, but the points could represent the positions of any feature e.g. transcription start sites. We use simple point processes on ℝ_+ _consisting of a sequence of points, (*T *(*i*))_*i*∈ℕ_, where 0 ≤ *T *(1) <*T *(2) <⋯. The corresponding counting process is denoted by *N *and is given by

$$\begin{array}{cc} N(t)=\sum_{i\in \mathbb{N}}1_{\left(T(i)\leq t\right)}, & t\in {\mathbb{R}}_{+} \end{array}.$$

Since there is a one-to-one correspondence between the point process and the corresponding counting process, the point process will simply be denoted by *N*.

The best-known point process is the Poisson process, for which points occur completely at random. For a homogeneous Poisson process, the points occur with a constant intensity (rate), *λ*, such that the mean number of points in an interval of length *l *is *λl*. For a general point process *N*, points do not occur on the line completely at random. At a given position *t*, information about previous points is contained in the history of the process. Generally, the intensity for the point process is dependent on the history before *t*. The intensity is a generalized form of the hazard function known from survival analysis, and a large intensity at a given position means that there is a relatively large probability of a point occurring immediately after that position.

A point process on the line can be uniquely specified by defining the intensity process, *λ*(*t*), *t ≥ *0 [26]. The expected number of points for the process between *t *and *t *+ *δ *is then approximately *δλ*(*t*) for small *δ*. In the homogeneous Poisson case, *δ λ*(*t*) = *δλ*is the actual expected value. The form of the intensity can be specified in a number of ways and can depend on covariates or other processes.

A marked point process is a simple point process with marks in a set K. Here, we assume that the set is finite, K = {1, 2,..., *K*}. More specifically, in our context, K is a collection of TREs. The marked point process *N *consists of both points and marks, (*T *(*i*), *K*(*i*)), *i *= 1,..., *n*, where *T *(*i*) ∈ ℝ_+ _and *K*(*i*) ∈ K. For C⊂K, the part of the point process with marks in C, $N_{C}$, is again a simple point process, $N_{C}(t)=\sum_{\mathbb{N}}1_{\left(T(i)\leq t,K(i)\in C\right)}$. In particular, the sequence of points with marks equal to *k*, *N_k_*, is a simple point process for each *k *∈ K. The collection of simple point processes for all *k *∈ K. can be viewed as a multivariate point process associated with the marked point process.

The history of a marked point process contains information about both the location of points and the type of mark at each point.

#### The likelihood function

When the marked point process is specified by a family of parameterized intensities, $\lambda_{\theta_{k}},\;{\left(\theta_{k}\right)}_{k=1,...,K}\in \Theta$, it is possible to write down the log-likelihood function for an observation of the process, ${\left(t_{1},...,t_{n_{k}}\right)}_{k=1,...,K}$, on *C *⊆ ℝ:

$$l(\theta )=\sum_{k=1}^{K}\left(\sum_{j=1}^{n_{k}}\log\lambda_{\theta_{k}}(t_{j})-\int_{C}\lambda_{\theta_{k}}(s)ds\right)$$

(see [26], p. 251). In general, there is no closed form for the maximum likelihood estimate and the likelihood function has to be optimized numerically.

Given *M *independent observations, ${\left\{t_{1}^{\left(i\right)},...,t_{n}^{\left(i\right)}\right\}}_{i=1,...,M}$, of a marked point process on {*C_i _*⊆ ℝ}_*i *= 1,..., *M*_, the log-likelihood function is the sum of the individual log-likelihood functions above:

$$l(\theta )\;=\;\sum_{i=1}^{M}\left(\sum_{k=1}^{K}\left(\sum_{j=1}^{n_{ki}}\log\lambda_{\theta_{k}}^{\left(i\right)}(t_{j}^{\left(i\right)})-\int_{C_{i}}\lambda_{\theta_{k}}^{\left(i\right)}(s)ds\right)\right),$$

where $\lambda_{\theta_{k}}^{\left(i\right)}$ is the intensity for the *i*th realization of the point process with mark *k*. The differencies in intensity can be due to different covariates for the individual realizations.

In our analysis, the *i*th realization is the *i*th chromosome in the mouse embryonic stem cell data or the *i*th ENCODE region, and the mark *k *is the TRE *k*. In our case, the different covariates are the baseline intensities and the two histone modifications that occur at different sites over the ENCODE sequences. The intensity for an occurrence of a TRE at a given sequence and a given site also depends on previous occurrences of the TREs and can therefore be larger or smaller than it would be without these previous occurrences.

Interchanging the first two sums in the formula for the log-likelihood function yields a sum of *K *likelihood functions, one for each marginal process. If the *K *likelihood functions do not share any parameters, as is the case for the Hawkes model considered below, it is possible to maximize the log likelihood function by maximizing each of the *K *terms separately.

#### Multivariate nonlinear Hawkes process

Our setup consists of multiple observations of point processes within bounded intervals, [*a_i_*, *b_i_*] ⊂ [*a*, *b*], *i *= 1,..., *M *(the mouse chromosomes or the ENCODE regions). We choose a log-linear parameterization of the intensity, $\lambda_{\theta_{k}}^{\left(i\right)}$, for process *k *in realization *i*,

$$\log\lambda_{\theta_{k}}^{\left(i\right)}(t)={\left(\alpha^{(i)k}\right)}^{T}X_{i}(t)+\sum_{m=1}^{K}\int_{\left[a,t\right]}h_{\beta }^{mk}(t-s)N_{m}^{\left(i\right)}(ds).$$

The *X_i_*s are covariates for the *i*th realization and are possibly position-dependent. $N_{m}^{\left(i\right)}$ is the point process for mark *m *in sequence *i*, and *α*^(*i*)*k *^is a parameter vector. Included in *X_i _*is a constant such that the first element of *α*^(*i*)*k *^is the logarithm of the baseline intensity for sequence *i*. For the mouse embryonic stem cell data, all sequences are assumed to have the same baseline intensity. For both data sets, the other elements of *α*^(*i*)*k *^do not vary with *i*. One advantage of using the log-linear parameterization is that the intensity automatically becomes positive, as is required. However, there is a potential technical problem with the specification, as it can lead to point processes that explode, i.e. point processes for which infinitely many points can occur in a bounded region. For simulation purposes, we resolve the problem by switching to a linear relation for large intensities, but we retain the log-linear specification for the estimation. This model is a special case of the multivariate nonlinear Hawkes model described in [27].

The $h_{\beta }^{mk}$-functions represent the effect of the occurrence of points of type *m *on subsequent points of type *k*. From the expression for the intensity $\lambda_{\theta_{k}}^{\left(i\right)}$, we see that if we define the function

$$g_{m,k}(s)=e^{h_{\beta }^{mk}(s)},$$

then if a point of type *m *occurs at position *s*, this affects the intensity for occurrence of a point of type *k *at position *t *>*s *by a factor *g_m, k_*(*t *- *s*). The major null hypotheses investigated in this paper are whether these fold-changes of the intensity are equal to 1. Moreover, we consider only covariate processes with values in {0, 1}. Defining

$$\gamma_{j}^{k}=e^{\alpha_{j}^{k}}$$

we observe that $\gamma_{j}^{k}$ is the fold-change of the intensity due to the *j*th covariate process being equal to 1. 

In principle, the $h_{\beta }^{mk}\text{s}$ could be arbitrary functions, in which case the parameter space would be infinitely dimensional. Here, we choose to model the $h_{\beta }^{mk}$-functions as linear combinations of spline functions,

$$h_{\beta }^{mk}(t)={\left(\beta^{mk}\right)}^{T}B(t)=\sum_{l=1}^{d_{\beta }}\beta_{l}^{mk}B_{l}(t),$$

where the *β^mk^*s are parameter vectors, and the B*_l_*s are cubic B-spline basis functions, such that $h_{\beta }^{mk}$ is a cubic spline [28].

The value of the largest knot gives the maximum range within which we will be able to detect dependencies with the method and hence must be chosen carefully. The number of knots determines how detailed the description of the dependencies can be. Choosing too many knots will cause the model to be over-fitted. To select the placement and number of knots, we conducted 2 pilot studies. One was based on an analysis of the occurrences of three TREs on one chromosome of the mouse genome and the other was based on an analysis of the occurrences of three TREs from the ENCODE data in the pilot ENCODE regions. These pilot studies suggested that 8 equidistant knots in the range -400 to 1000 base pairs was computationally feasible while still sufficiently flexible for the current analysis.

We have established a fully parameterized specification of our model and, given a realization of a point pattern, we estimate the parameter values by using maximum likelihood methods. This is implemented in the R package ppstat, given in Additional file 9 and 10. In Additional file 11 some details on the computation of the log-likelihood function above and its first and second derivatives are presented. The estimation is done using the optim function in R with optimization method "BFGS" or "L-BFGS-B" [29]. These optimization methods are quasi-Newton methods and require the computation of gradients, which is also implemented. In our parametrization, the log-likelihood function is concave, see [26], p. 235, which ensures that if the optimization algorithm converges to a local maximum, this will actually be the global maximum and therefore the maximum likelihood estimate.

With a probability tending to one, the likelihood function has one local maximum and the maximum likelihood estimates are normally distributed with mean equal to the true mean and a covariance matrix that can be estimated as the inverse of the matrix of second-order partial derivatives of the negative log-likelihood function, see [30].

The properties of the likelihood function enable us to construct pointwise confidence intervals for the *g*-functions and to carry out likelihood ratio tests for the covariates and covariate processes. In particular, we can test the hypothesis that one of the *g*-functions is equal to 1. The pointwise confidence intervals for the *h*-functions are calculated as

$$h_{\hat{\beta }}^{mk}(t)\pm z_{0.975}\sqrt{B{\left(t\right)}^{T}{\hat{\Sigma }}_{mk}B(t)},$$

where ${\hat{\Sigma }}_{mk}$ is the estimated covariance matrix for the estimated parameters, ${\hat{\beta }}^{mk}\;$, *B*(*t*) is the vector of values for the spline bases at *t*, and *z*_0.975 _is the 97.5% quantile for the normal distribution. These confidence intervals are transformed using the exponential function to yield confidence intervals for the *g*-functions. 

The likelihood ratio test statistic for *H*_0 _: *g_m, k _*= 1 is *Q *= 2(*l*_1 _- *l*_0_), where *l_0 _*is the value of the maximized log-likelihood function for the model with *g_m, k _*= 1, and *l*_1 _is the value of the maximized log-likelihood function for the full model. In this case, the null distribution for the test statistics can be approximated by the *χ*^2 ^distribution with 4 degrees of freedom.

#### Local independence

In the context of multivariate point processes there is a concept of local independence between the *K *simple point processes. A formal definition of local independence between point processes is given in [9]. An informal definition is as follows: For *A*, *B*, *C *disjoint subsets of K; the process *N_B _*is locally independent of *N_A _*given the history of *N_B _*and *N_C _*if the intensity for *N_B _*is the same when we only have information about events with marks in *B *⋃ *C *as when we have information about events with marks in *A *⋃ *B *⋃ *C*. More concretely, for the Hawkes processes we consider, testing *H*_0 _: *g_m, k _*= 1 is equivalent to testing whether *N_k _*is locally independent of *N_m _*given $N_{K\backslash m}$. As an illustration, consider the case where *g_m, k _*= 1. When this is true the intensity for *N_k _*does not depend on occurrences of points for *N_m_*, and *N_k _*is therefore locally independent of *N_m _*given $N_{K\backslash m}$. On the other hand, if *N_k _*is locally independent of *N_m _*given $N_{K\backslash m}$, then the intensity for *N_k _*does not depend on the locations of points for *N_m_*, and we have *g_m, k _*= 1. We refer to [9] for details.

#### Clustering

To find groups of TREs that are likely to act together in the regulation of genes, we propose a simple cluster analysis based on the results from the multivariate analysis. We consider a hierarchical cluster analysis based on the $h_{\beta }^{mk}$-functions, where we use the integral of the absolute value of the functions,

$$H_{\beta }^{mk}=\int_{0}^{1000}|h_{\beta }^{mk}(t)|dt$$.

as a measure of similarity. Euclidean distance is used to create the distance matrix and a hierarchical clustering procedure is applied based on the Ward method [31]. This method produces groups that are as homogeneous as possible since it is based on an error sum of squares criterion. The resulting clusters are groups of TREs such that $H_{\beta }^{mk}$ is relatively large for *m *and *k *within the same group and small for *m *and *k *in different groups. For instance, if three TREs frequently co-occur and generally do not co-occur with other TREs, then the |$h_{\beta }^{mk}$| will be large for these three TREs (*m *and *k *in the set of the three TREs) but small if *m *or *k *is not one of the three TREs. Consequently, the three TREs are likely to be in the same cluster. We take the absolute value of $h_{\beta }^{mk}(t)$ above to prevent positive and negative values of the function from cancelling out in the integration. As a result, two TREs can also end up in the same cluster if the corresponding transcription factors repress the binding of one another.

#### Computational considerations

The central computations in the current implementation involve a large, sparse model matrix. The number of columns in the matrix is of the order *O*(*k*_0_*n*_0_), where *k*_0 _is the number of spline basis functions and *n*_0 _is the number of TREs analyzed. The number of rows is of the order *O*(*ML/r*) where *M *is the total number of TRE observations, *L *is range of dependencies (here *L *= 1000), and *r *is the resolution. The finest resolution in our analyses is 1 bp, which was used for the ENCODE data. The largest model we have estimated is a genome-wide model for the mouse embryonic stem cell data including all 15 TREs from [10] with resolution *r *= 10 bp. To do this, we used approximately 30 GB RAM. Once the model matrix is computed the actual computation of the log-likelihood and gradient, and thus the optimization, is comparatively fast, and the limiting factors are currently the time for computation of the model matrix and the associated memory consumption.

## List of abbreviations

PCA: principal component analysis; TRE: transcriptional regulatory element; BRG1: SWI/SNF related, matrix associated, actin dependent regulator of chromatin, subfamily a, member 4; CEBPE: CCAAT/enhancer binding protein (C/EBP), epsilon; CTCF: CCCTC-binding factor (zinc finger protein); c-myc: myelocytomatosis oncogene; E2f1: E2F transcription factor 1; H3K27me3 (H3K27T): Histone H3 tri-methylated lysine 27; H4Kac4 (HisH4): Histone H4 tetra-acetylated lysine; Nanog: Nanog homeobox; n-myc: v-myc myelocytomatosis viral related oncogene, neuroblastoma derived; Oct4: POU domain, class 5, transcription factor 1; p300: E1A binding protein p300; POL2: polymerase (RNA) II (DNA directed) polypeptide A, 220 kDa; PU1: Spleen focus forming virus proviral integration oncogene; RARA (RARecA): Retinoic Acid Receptor-Alpha; SIRT1; sirtuin (silent mating type information regulation 2 homolog) 1; Smad1: MAD homolog 1; Zfx: zinc finger protein X-linked; Sox2: SRY-box containing gene 2; Stat3: signal transducer and activator of transcription 3; Suz12: suppressor of zeste 12 homolog.

## Authors' contributions

LC developed, with assistance from NRH and OW, the multivariate Hawkes model as used in the paper. LC and NRH implemented the model. LC carried out the data analysis and wrote a first draft of the paper. AS assisted with obtaining, selecting and interpreting the ENCODE data and with the biological implications of the data analysis. All authors collaborated on the interpretation of the data analysis. LC and NRH wrote, with assistance from AS, the final version of the paper. All authors read and approved the final manuscript.

## Supplementary Material

Additional file 1 **Mouse embryonic stem cell data - Part I**. Coordinates of loci bound by Nanog, Oct4, Sox2, E2f1, Smad1, Zfx, c-myc, n-myc and Stat3. Click here for file

Additional file 2 **Mouse embryonic stem cell data - Part II**. Coordinates of loci bound by p300 and Suz12. Click here for file

Additional file 3 **ENCODE pilot data - hr00**. Affymetrix ChIP-chip sites for the ENCODE pilot project to time hr00 with chromosome number, start position and end position for the enriched regions. Click here for file

Additional file 4 **ENCODE pilot data - hr02**. Affymetrix ChIP-chip sites for the ENCODE pilot project to time hr02 with chromosome number, start position and end position for the enriched regions. Click here for file

Additional file 5 **ENCODE pilot data - hr08**. Affymetrix ChIP-chip sites for the ENCODE pilot project to time hr08 with chromosome number, start position and end position for the enriched regions. Click here for file

Additional file 6 **ENCODE pilot data - hr32**. Affymetrix ChIP-chip sites for the ENCODE pilot project to time hr32 with chromosome number, start position and end position for the enriched regions. Click here for file

Additional file 7 **ENCODE pilot data - The 44 pilot regions**. The locations and names of the 44 ENCODE pilot regions. Click here for file

Additional file 8 **Illustration of the occurrences of TREs in the ENCODE pilot regions**. Illustration of the pilot ENCODE regions with the occurrences of the 10 TREs marked as point processes. Click here for file

Additional file 9 **Information on installation of the R package ppstat**. A PDF file of the web page for the R package ppstat (as of 12 August 2010) including information on installation. Click here for file

Additional file 10 **Source code for the R package ppstat**. Source code for the R package ppstat. Click here for file

Additional file 11 **Note on the computations of the log-likelihood function**. Note on the computations of the log-likelihood function and its first and second derivatives. Click here for file
