# Supplementary material for: Multivariate Hawkes process models of the occurrence of regulatory elements
Source: BMC Bioinformatics. 2010 Sep 9;11:456. doi: 10.1186/1471-2105-11-456 (PMC2949889; doi:10.1186/1471-2105-11-456)
Supplement: Additional file 8 — Illustration of the occurrences of TREs in the ENCODE pilot regions. Illustration of the pilot ENCODE regions with the occurrences of the 10 TREs marked as point processes. [file 1471-2105-11-456-S8.PDF]

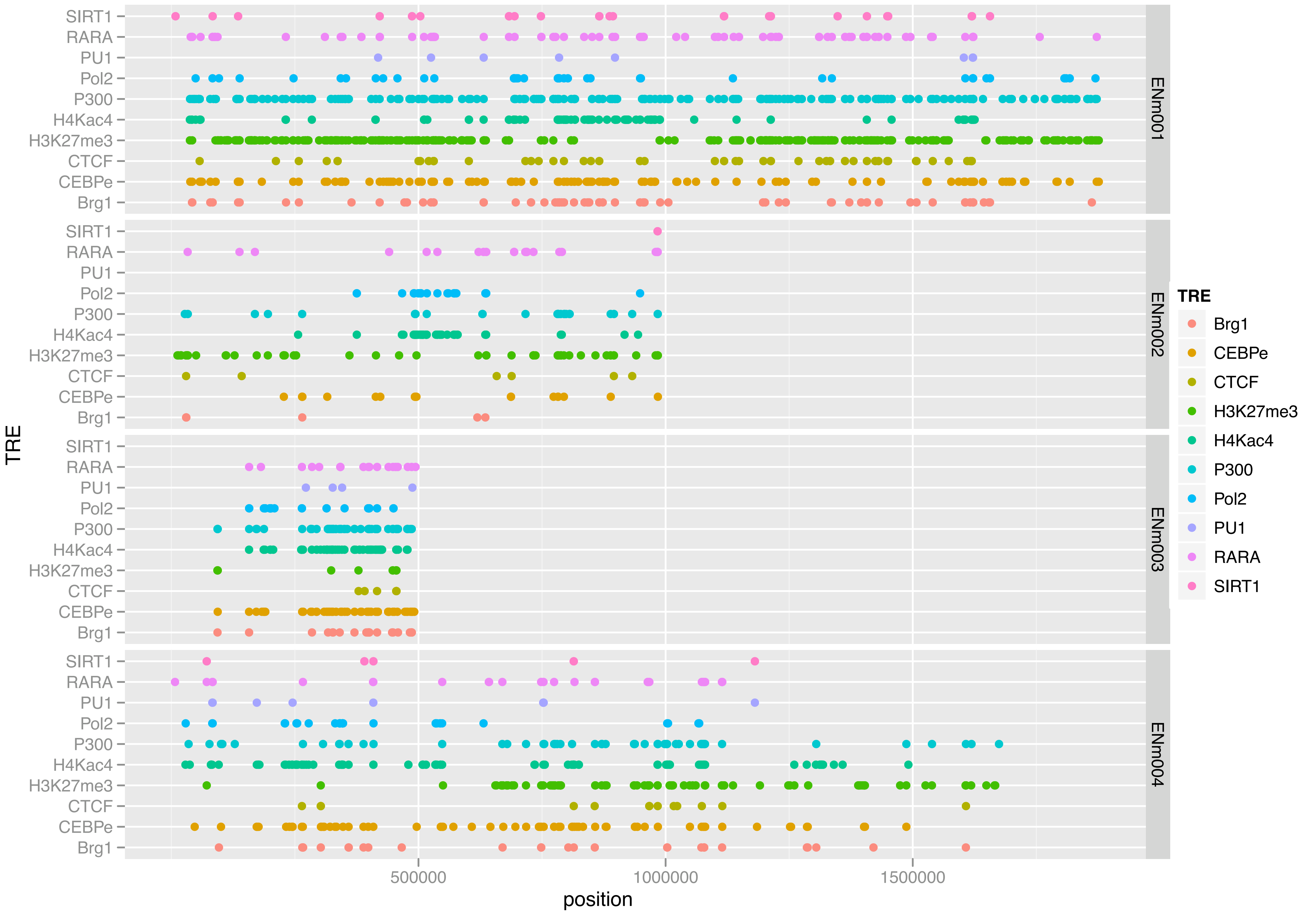

SIRT1
RARA
PU1
Pol2
P300
H4Kac4
H3K27me3
CTCF
CEBPe
Brg1
ENm001
ENm002
ENm003
ENm004
TRE
500000
1000000
1500000
position
TRE
Brg1
CEBPe
CTCF
H3K27me3
H4Kac4
P300
Pol2
PU1
RARA
SIRT1

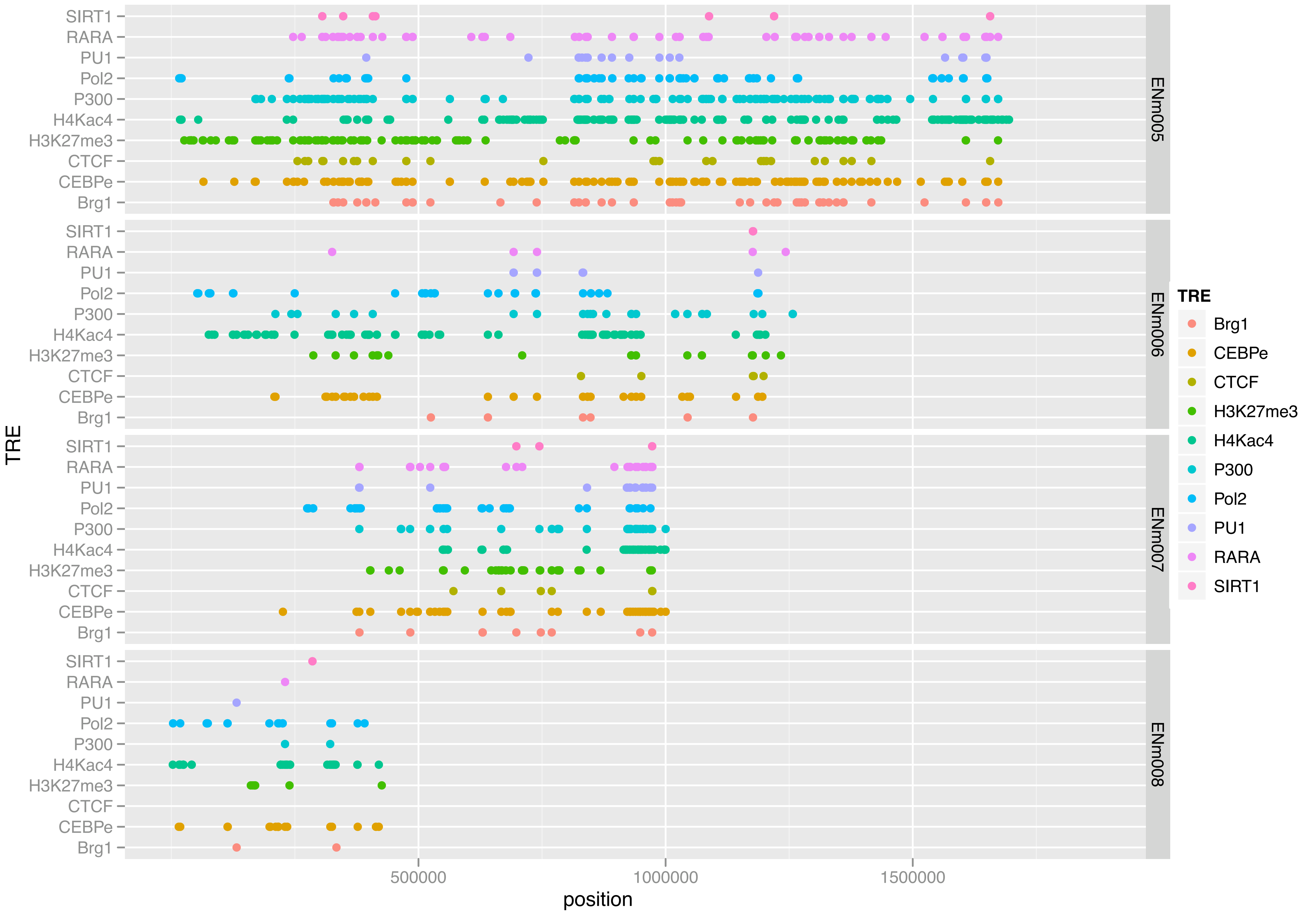

SIRT1
RARA
PU1
Pol2
P300
H4Kac4
H3K27me3
CTCF
CEBPe
Brg1
TRE
position
500000
1000000
1500000
ENm005
ENm006
ENm007
ENm008
TRE
Brg1
CEBPe
CTCF
H3K27me3
H4Kac4
P300
Pol2
PU1
RARA
SIRT1

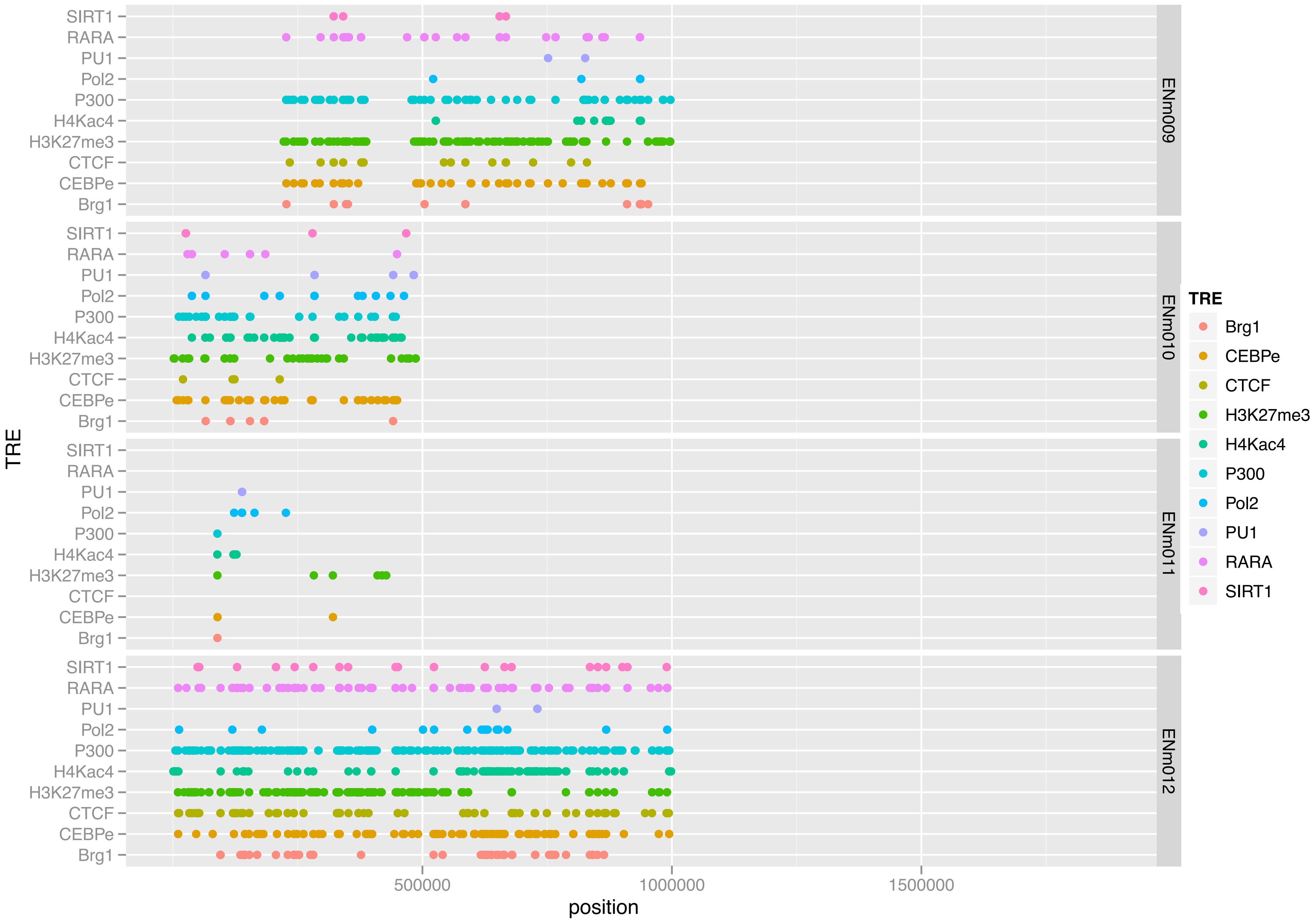
SIRT1
RARA
PU1
Pol2
P300
H4Kac4
H3K27me3
CTCF
CEBPe
Brg1
ENm009
ENm010
ENm011
ENm012
TRE
500000
1000000
1500000
position
TRE
Brg1
CEBPe
CTCF
H3K27me3
H4Kac4
P300
Pol2
PU1
RARA
SIRT1

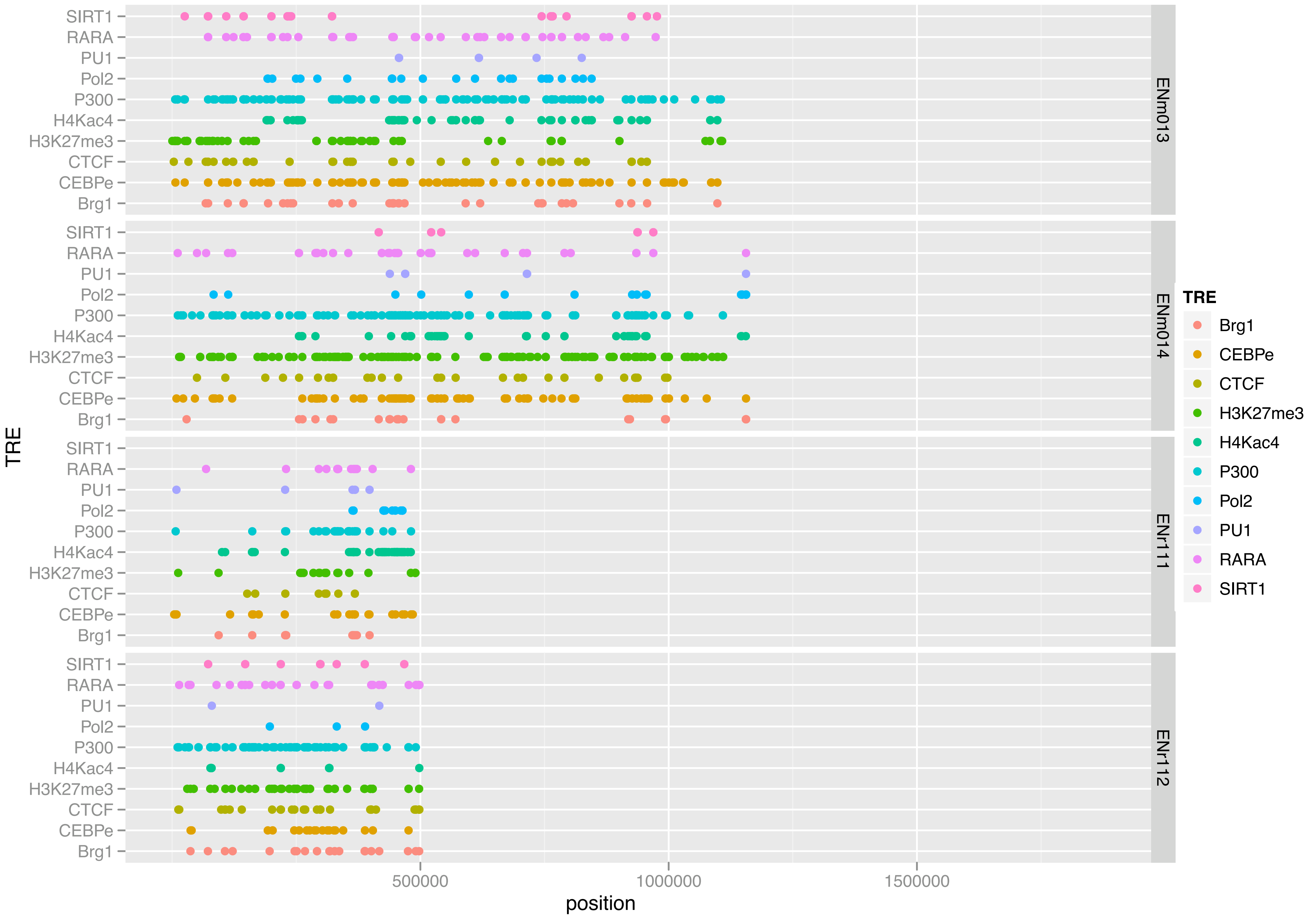

SIRT1
RARA
PU1
Pol2
P300
H4Kac4
H3K27me3
CTCF
CEBPe
Brg1
ENm013
ENm014
ENr111
ENr112
TRE
500000
1000000
1500000
position
TRE
Brg1
CEBPe
CTCF
H3K27me3
H4Kac4
P300
Pol2
PU1
RARA
SIRT1

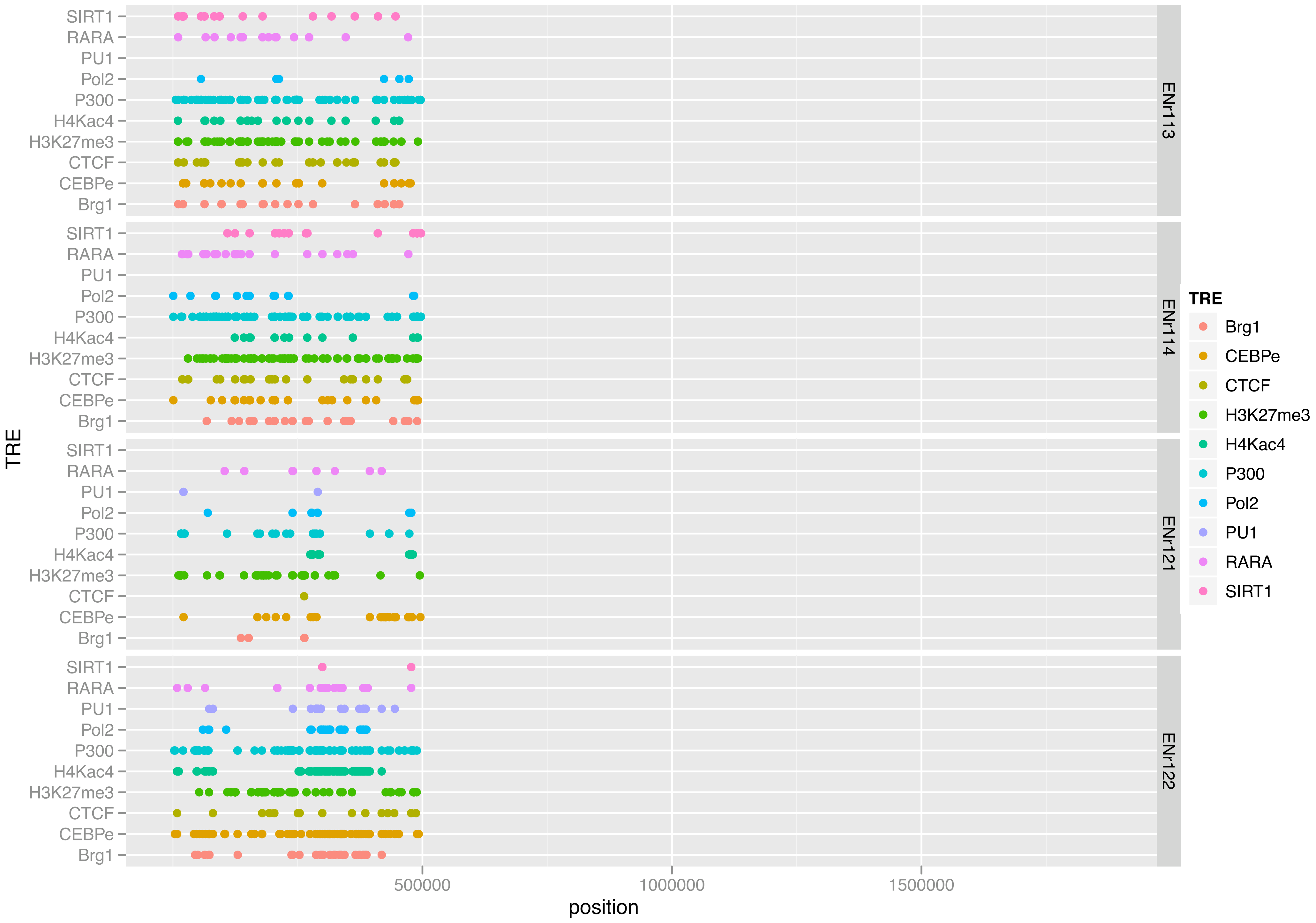
SIRT1
RARA
PU1
Pol2
P300
H4Kac4
H3K27me3
CTCF
CEBPe
Brg1
ENr113
ENr114
ENr121
ENr122
TRE
500000
1000000
1500000
position
TRE
Brg1
CEBPe
CTCF
H3K27me3
H4Kac4
P300
Pol2
PU1
RARA
SIRT1

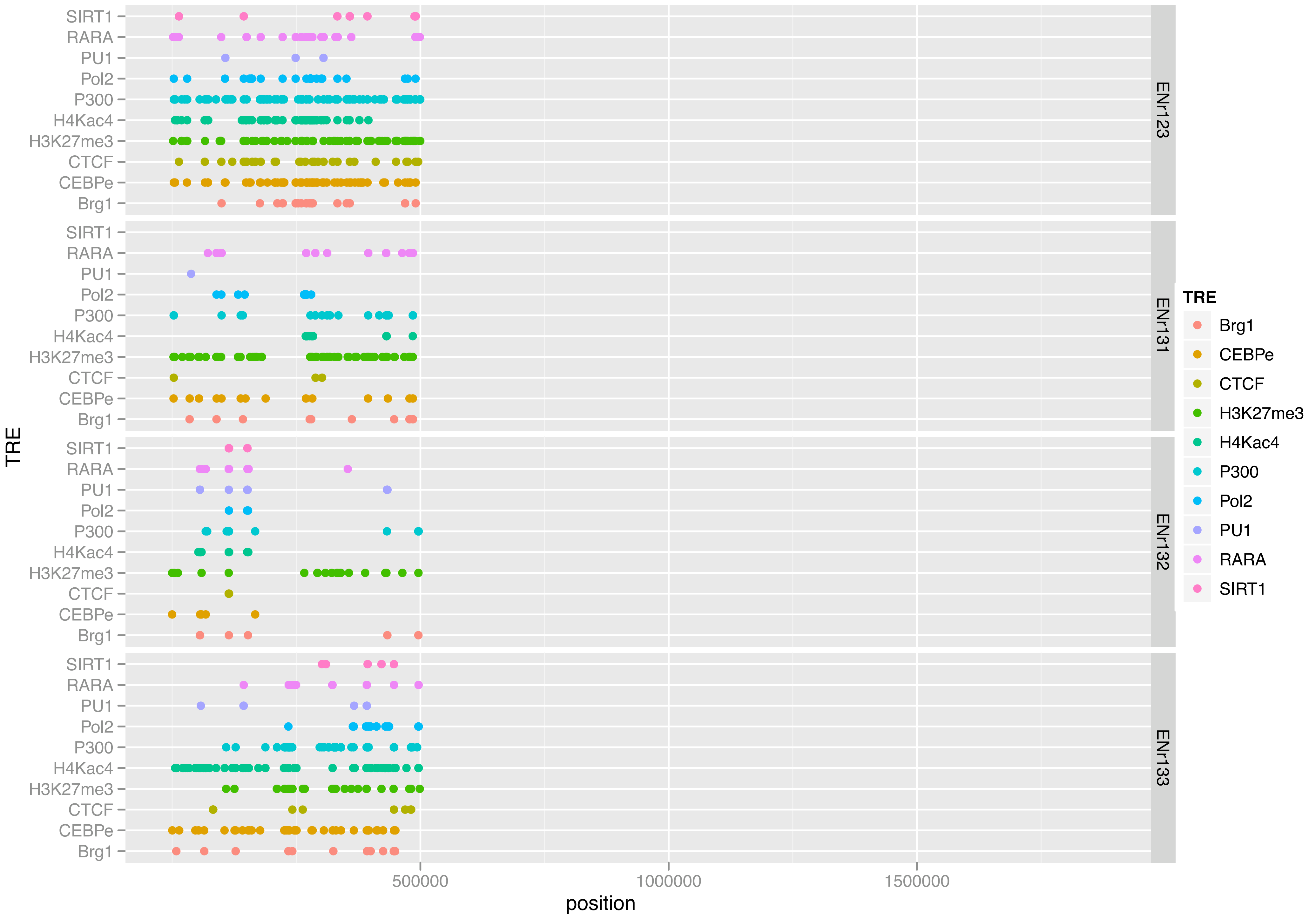

SIRT1
RARA
PU1
Pol2
P300
H4Kac4
H3K27me3
CTCF
CEBPe
Brg1
ENr123
ENr131
ENr132
ENr133
TRE
500000
1000000
1500000
position
TRE
Brg1
CEBPe
CTCF
H3K27me3
H4Kac4
P300
Pol2
PU1
RARA
SIRT1

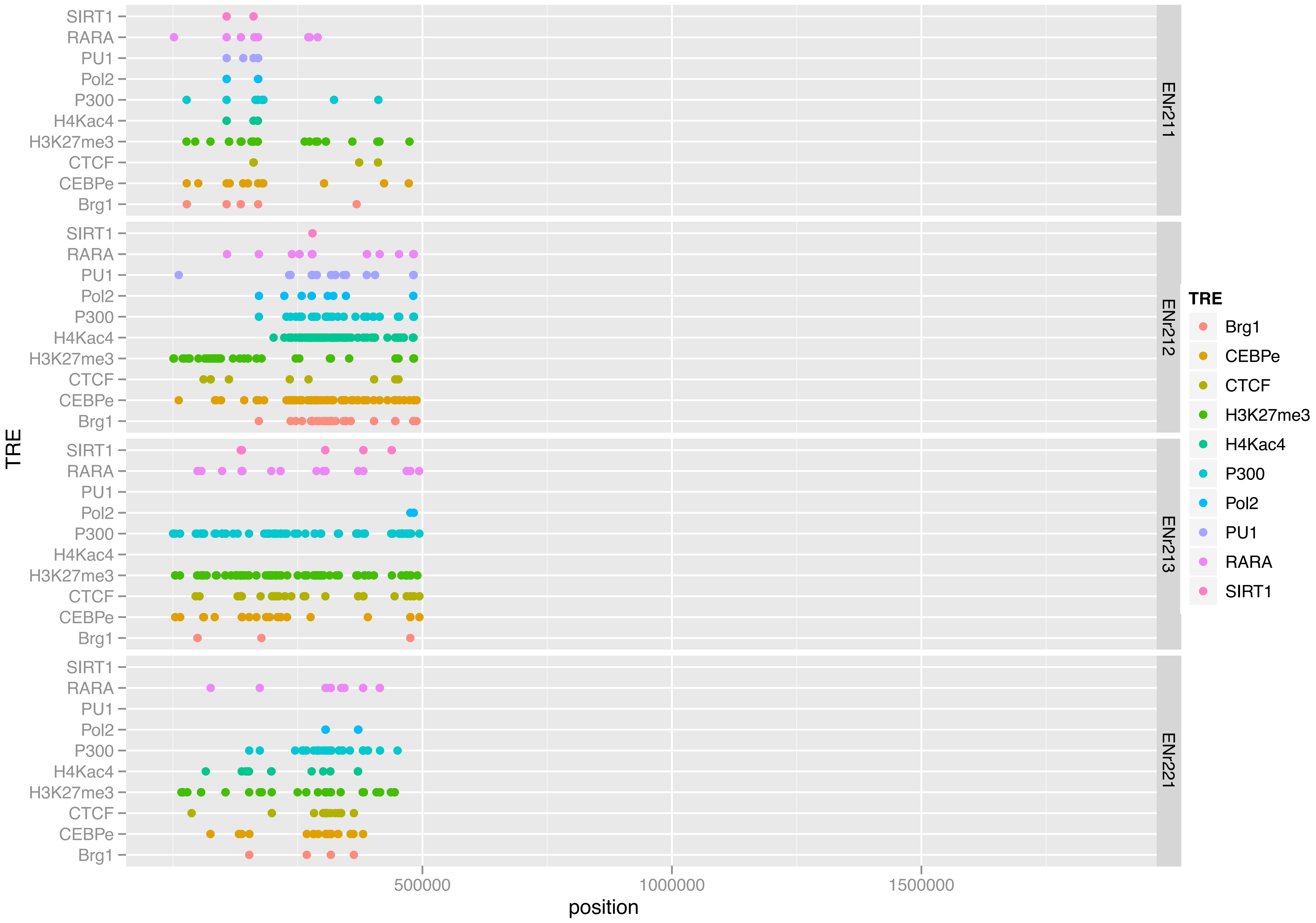

SIRT1
RARA
PU1
Pol2
P300
H4Kac4
H3K27me3
CTCF
CEBPe
Brg1
ENr211
ENr212
ENr213
ENr221
TRE
500000
1000000
1500000
position
TRE
Brg1
CEBPe
CTCF
H3K27me3
H4Kac4
P300
Pol2
PU1
RARA
SIRT1

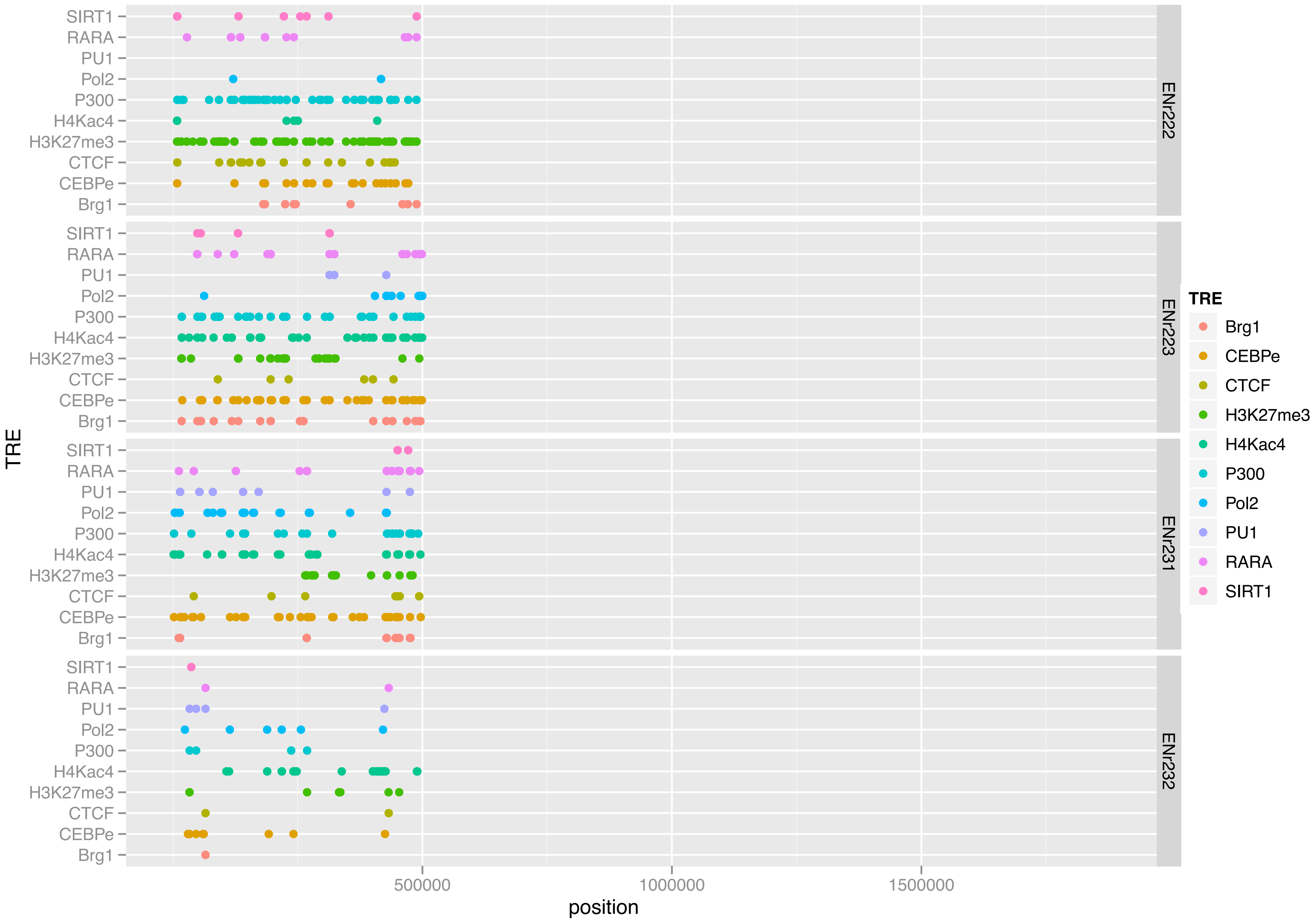

SIRT1
RARA
PU1
Pol2
P300
H4Kac4
H3K27me3
CTCF
CEBPe
Brg1
ENr222
ENr223
ENr231
ENr232
TRE
500000
1000000
1500000
position
Brg1
CEBPe
CTCF
H3K27me3
H4Kac4
P300
Pol2
PU1
RARA
SIRT1

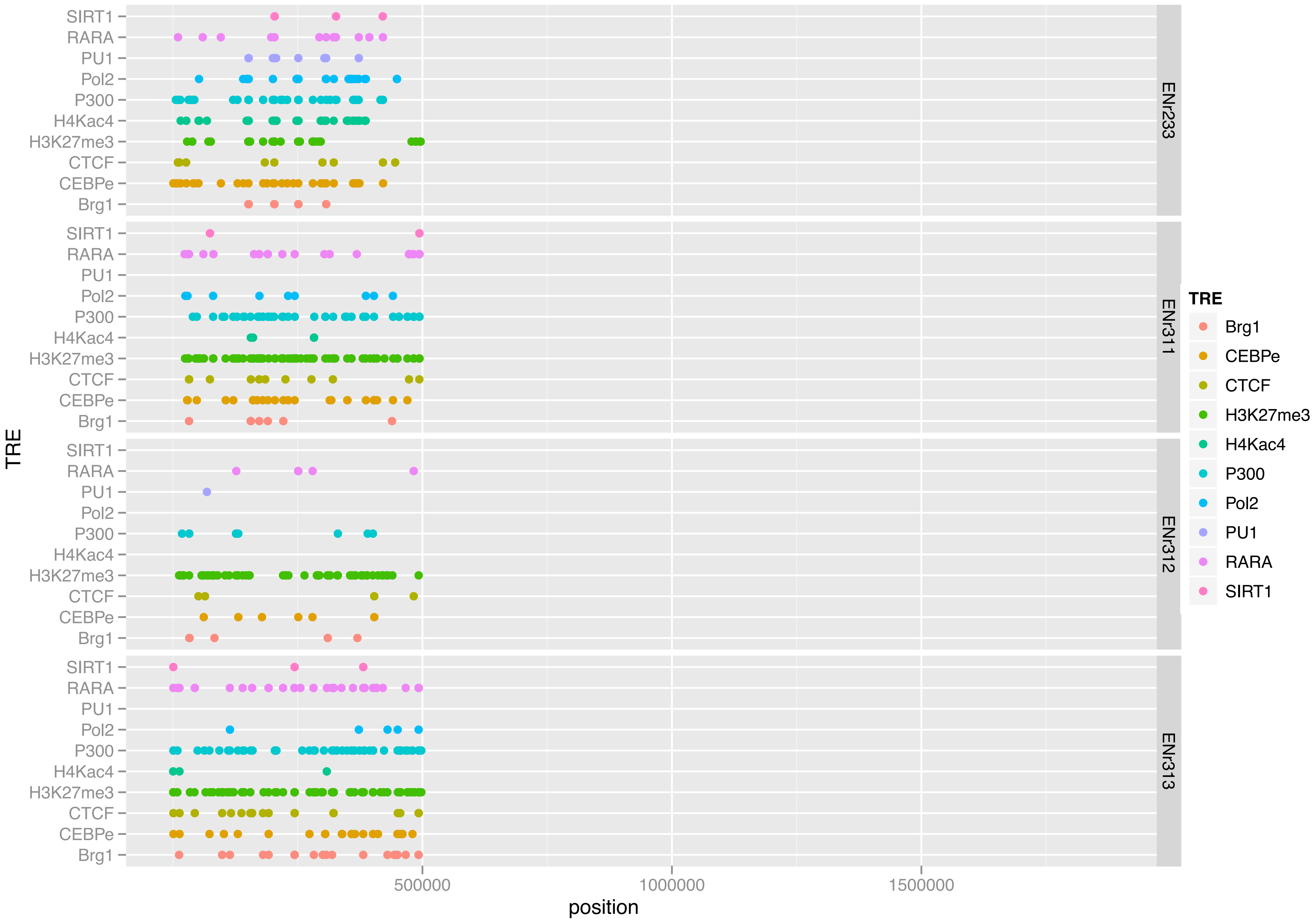

SIRT1
RARA
PU1
Pol2
P300
H4Kac4
H3K27me3
CTCF
CEBPe
Brg1
ENr233
ENr311
ENr312
ENr313
TRE
500000
1000000
1500000
position
TRE
Brg1
CEBPe
CTCF
H3K27me3
H4Kac4
P300
Pol2
PU1
RARA
SIRT1

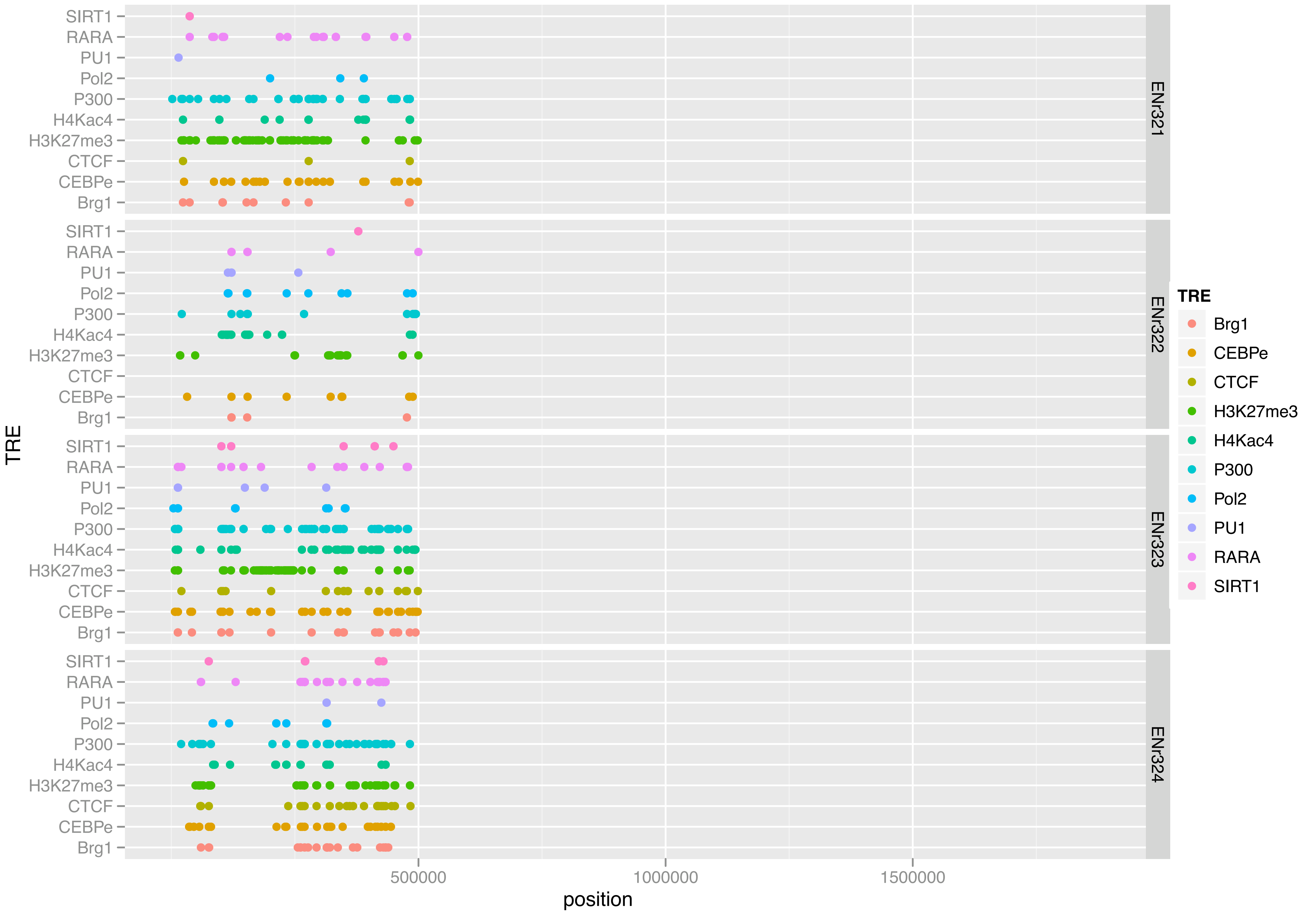

SIRT1
RARA
PU1
Pol2
P300
H4Kac4
H3K27me3
CTCF
CEBPe
Brg1
TRE
ENr321
ENr322
ENr323
ENr324
500000
1000000
1500000
position
TRE
Brg1
CEBPe
CTCF
H3K27me3
H4Kac4
P300
Pol2
PU1
RARA
SIRT1

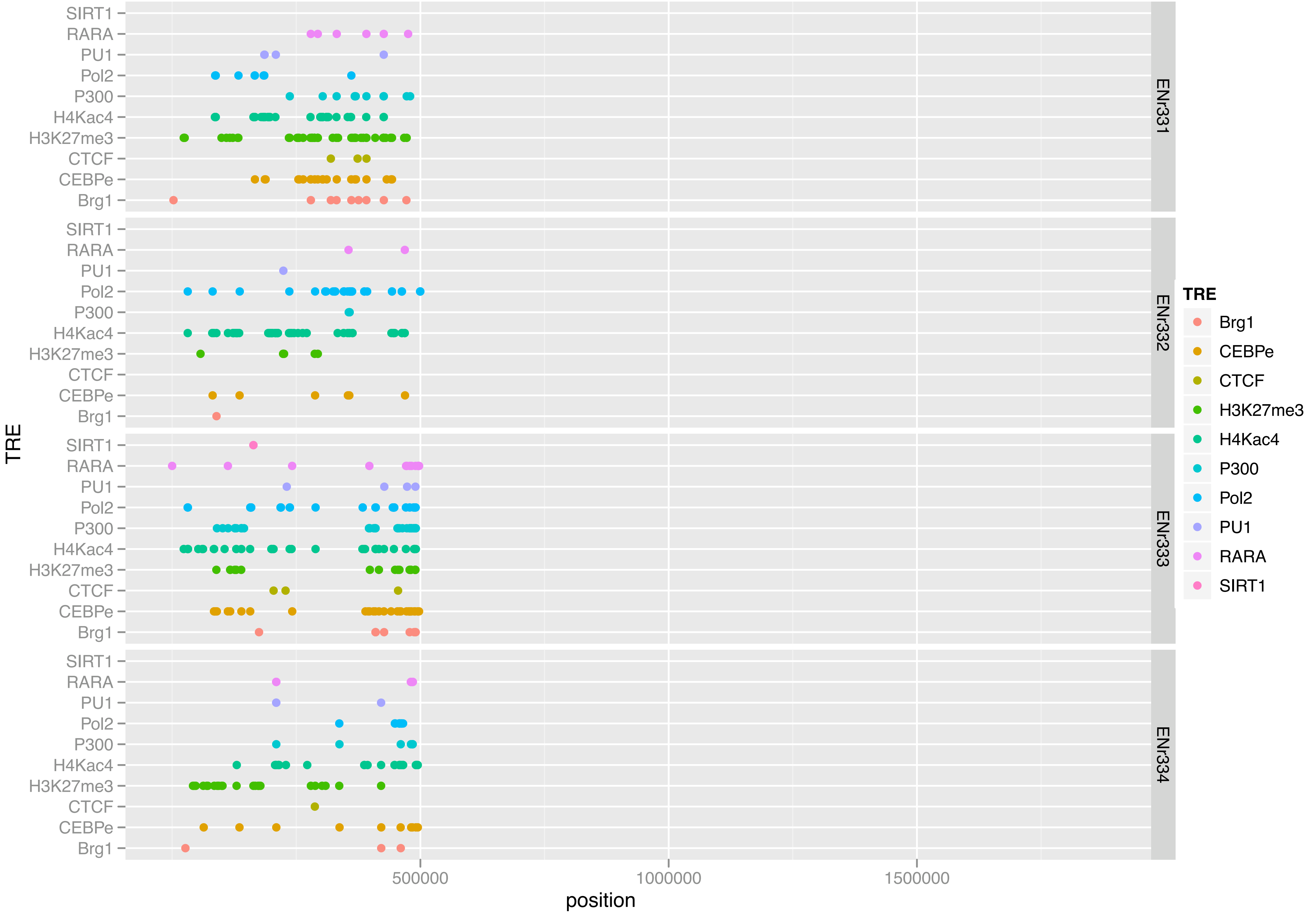

SIRT1
RARA
PU1
Pol2
P300
H4Kac4
H3K27me3
CTCF
CEBPe
Brg1
ENr331
ENr332
ENr333
ENr334
TRE
500000
1000000
1500000
position
TRE
Brg1
CEBPe
CTCF
H3K27me3
H4Kac4
P300
Pol2
PU1
RARA
SIRT1
