# Supplementary material for: Multivariate Hawkes process models of the occurrence of regulatory elements
Source: BMC Bioinformatics. 2010 Sep 9;11:456. doi: 10.1186/1471-2105-11-456 (PMC2949889; doi:10.1186/1471-2105-11-456)
Supplement: Additional file 9 — Information on installation of the R package ppstat. A PDF file of the web page for the R package ppstat (as of 12 August 2010) including information on installation. [file 1471-2105-11-456-S9.PDF]

UNIVERSITY OF COPENHAGEN

Department of Mathematical Sciences

ppstat

The R-package **ppstat** is a beta-version of a forthcoming R-package that will also be available from CRAN for doing point process statistics for multivariate point processes (that is, marked point processes) on the line. The current beta-version comes with a data structure for storing point process and general stochastic process data, including several plotting facilities, and an implementation of the statistical methods for the multivariate Hawkes process.

License: GPLv3

Comments and bug reports

Other programs on my web page

Download

- **Download version 0.4** (source)

Older versions

- **Download version 0.3** (source)
- **Windows binary, R 2.9.2 build, version 0.3**

Installation and usage

- Install the package *ggplot2* from CRAN, e.g. by typing

  ```
  install.packages("ggplot2")
  ```

  in a running R session, or from the menu using the Mac or Windows GUI.

  - If you have downloaded the .tar.gz source file above (for Linux or Mac installation, for using the source file on Windows see Section 6.3.1 in the "R Installation and Administration" manual) and the downloaded file is called *ppstat_version.tar.gz* and is located in *path* type

    ```
    $ R CMD INSTALL  path/ppstat_version.tar.gz
    ```

    at the shell prompt to install the package.

  - If you have downloaded the Windows binary .zip file install the package from the menu:

    ```
    Packages/Install package(s) from local zip files
    ```

  You will need to have R version >= 2.9.0 installed, see The R project for further informations.

- To load the package in R type

  ```
  library(ppstat)
  ```

  in a running R session.

Getting help

The package is still under development and in an experimental phase. Some useful informations can be found from the help pages, try e.g.

```
?"ppstat-package"
?glppm
```

To use the package you will have to construct an object of class ProcessDatacontaining your data and use glppmto fit the model using a formula specification of the model. The demo exampleshows some examples. To see the results of running the demo type

```
demo(examples)
```

and to get the location of the *examples.R* file type

```
system.file("demo","examples.R",package="ppstat")
```

Niels Richard Hansen

Last modified: Thu Sep 23 16:03:29 2009
