# Supplementary material for: Multivariate Hawkes process models of the occurrence of regulatory elements
Source: BMC Bioinformatics. 2010 Sep 9;11:456. doi: 10.1186/1471-2105-11-456 (PMC2949889; doi:10.1186/1471-2105-11-456)
Supplement: Additional file 11 — Note on the computations of the log-likelihood function. Note on the computations of the log-likelihood function and its first and second derivatives. [file 1471-2105-11-456-S11.PDF]

Computational details for ppstat

Niels Richard Hansen[1], *University of Copenhagen*

The core computational problem in the use of point processes for statistical modeling is the optimization of the minus-log-likelihood function, which is given as

$$l(\theta) = \sum_{j=1}^{n} \log \lambda_\theta(t_j) - \int_0^T \lambda_\theta(s) \mathrm{d}s$$

where $0 < t_1 < t_2 < \ldots < t_n < T$ are observations and λ_θ is a parameterized family of intensities. Typically $\theta \in \Theta \subseteq \mathbb{R}^p$. For the Hawkes family of generalized linear point process models in `ppstat` we consider situations where

$$\lambda_\theta(t) = \varphi\left(\alpha^T X(t) + \sum_{m=1}^{K} \sum_{i=1}^{n(m)} h^m_{\beta^m}(t - s^m_i)\right)$$

where $\varphi : \mathbb{R} \to \mathbb{R}$ is a given function,

$$\theta = \begin{pmatrix} \alpha \\ \beta^1 \\ \vdots \\ \beta^K \end{pmatrix}$$

and $s^m_1 < \ldots < s^m_{n(m)}$ for $m = 1, \ldots, K$ are observations of point processes (one of these sets of points could be the t_i observations above). The process $X(t)$ is an auxiliary, $d(0)$-dimensional observed processes – observed at least discretely. The processes

$$\sum_{i=1}^{n(m)} h^m_{\beta^m}(t - s^m_i)$$

are linear filters using the (parameterized) filter function $h^m_{\beta^m}$, which are given via a basis expansion

$$h^m_{\beta^m}(t) = (\beta^m)^T B(t) = \sum_{l=1}^{d(m)} \beta^m_l B_l(t),$$

and $\beta^m \in \mathbb{R}^{d(m)}$. Collecting these ingredients – and interchanging two sums – the intensity function can be written as

$$\lambda_\theta(t) \quad = \quad \varphi\left(\alpha^T X(t) + \sum_{m=1}^{K} \sum_{l=1}^{d(m)} \beta^m_l \sum_{i=1}^{n(m)} B_l(t - s^m_i)\right) = \varphi\left(\theta^T Z(t)\right)$$

[1]Postal address: Department of Mathematical Sciences, University of Copenhagen Universitetsparken 5, 2100 Copenhagen Ø, Denmark.
Email address: richard@math.ku.dk

with $Z(t)$ a process of dimension $p = d(0) + d(1) + \ldots + d(K)$. Each of the linear filter components

$$\sum_{i=1}^{n(m)} B_l(t - s_i^m) \tag{1}$$

are computable from the observations and the fixed choice of basis. The minus-log-likelihood function that we want to minimize reads

$$l(\theta) = \int_0^T \varphi\left(\theta^T Z(s)\right) \mathrm{d}s - \sum_{j=1}^{n} \log \varphi\left(\theta^T Z(t_j)\right).$$

The integral is not in general analytically computable. We discretize time to have a total of N time points and let Z denote the $N \times p$ matrix of the $Z(t)$-process values at the discretization points. With Δ the N-dimensional vector of interdistances from the discretization we arrive at the approximation of the minus-log-likelihood function that we seek to minimize:

$$l(\theta) \simeq \Delta^T \varphi\left(\theta^T Z\right) - \sum_{j=1}^{n} \log \varphi\left(\theta^T Z(t_j)\right).$$

We have used the convention that φ applied to a vector means coordinate-wise applications of φ. Using this expression a precomputation of the Z matrix will allow for a rapid computation of (the approximation to) l. The derivatives are likewise approximated as

$$Dl(\theta) \simeq [\Delta \circ \varphi'\left(\theta^T Z\right)]^T Z - \sum_{j=1}^{n} \frac{\varphi'\left(\theta^T Z(t_j)\right)}{\varphi\left(\theta^T Z(t_j)\right)} Z(t_j)^T$$

with $\circ$ the Hadamard (or coordinate-wise) matrix product and

$$D^2 l(\theta) \simeq Z^T [\Delta \circ \varphi''\left(\theta^T Z\right) \circ Z] - \sum_{j=1}^{n} \frac{\varphi''\left(\theta^T Z(t_j)\right) \varphi\left(\theta^T Z(t_j)\right) - \varphi'\left(\theta^T Z(t_j)\right)^2}{\varphi\left(\theta^T Z(t_j)\right)^2} Z(t_j) Z(t_j)^T.$$
